# PER2 interaction with HSP70 promotes cuproptosis in oral squamous carcinoma cells by decreasing AKT stability

**DOI:** 10.1038/s41419-025-07523-1

**Published:** 2025-03-20

**Authors:** Wenguang Yu, Shilin Yin, Hong Tang, Hengyan Li, Zhiwei Zhang, Kai Yang

**Affiliations:** Department of Oral and Maxillofacial Surgery, The First Affiliated Hospital of Chongqing Medical University, Chongqing, 400016 China

**Keywords:** Oral cancer, Head and neck cancer

## Abstract

Oral squamous cell carcinoma (OSCC) has a poor prognosis, with unclear mechanisms posing a challenge for the development of effective treatment strategies. Cuproptosis is a novel cell death mode that disrupts mitochondrial metabolism. Clarifying the mechanisms that regulate cuproptosis may provide important new insights to guide OSCC treatment. Here, we found that the biological clock gene *Period2* (*PER2*) was under-expressed in OSCC, with consequent inhibition of cellular cuproptosis, whereas it was overexpression of *PER2* in vitro and in vivo and promoted OSCC cellular cuproptosis. Mechanistically, PER2 bound to heat shock protein 70 (HSP70) through its C-terminal domain, subsequently reducing the interaction between HSP70 and AKT and leading to enhanced degradation of AKT ubiquitination, and promoting cuproptosis in OSCC cells by inhibiting the AKT pathway and upregulating DLAT, PDHB, and SLC31A1 expression. Activating transcription factor 3 (ATF3) is an upstream regulator of *PER2*, that binds to the −807 to −796 bp site of the *PER2* promoter. Overexpression of *ATF3* in vitro and in vivo is dependent on transcriptional activation of *PER2* and promotes cuproptosis in OSCC cells. The anti-tumor effect of ATF3 inducer 1-targeted upregulation of *PER2* combined with copper ionophore elesclomol (ES) was found to be significantly enhanced compared with that of monotherapy in an OSCC xenograft model. These findings reveal a critical role of ATF3-dependent regulation of cuproptosis by *PER2* in OSCC development, suggesting targeted upregulation of *PER2* or *ATF3* in combination to induce cuproptosis as a novel strategy to potentially improve the prognosis of OSCC patients.

## Introduction

Oral squamous cell carcinoma (OSCC) is among the most common head and neck cancers [1, 2] and has an increasing incidence [3, 4], with approximately 370,000 new cases of OSCC and more than 170,000 OSCC-related deaths per year worldwide [2]. OSCC is highly aggressive and has a poor prognosis. Owing to its unclear pathogenesis, it is usually treated with surgery, supplemented with radiotherapy, chemotherapy, and immunotherapy for patients with intermediate and advanced disease [5]. Despite great progress in these treatment modalities in recent decades, the overall 5-year survival rate of OSCC patients has remained at around 50% and has not shown significant improvements [4]. Therefore, in-depth study of the molecular mechanisms underlying the development of OSCC and development of novel and effective treatment modalities are crucial to improve the survival of OSCC patients.

In March 2022, Tsvetkov et al. [6] reported a newly discovered form of cell death that disrupts the mitochondrial tricarboxylic acid (TCA) cycle; this was termed cuproptosis. Cuproptosis occurs as a result of dysregulation of copper homeostasis, in which excess intracellular copper binds to lipoylated proteins in the mitochondrial TCA (e.g., the commonly lipoylated DLAT protein), resulting in oligomerization of these proteins; this, in turn, triggers loss of iron-sulfur cluster proteins and proteotoxic stress within mitochondria, leading to impaired mitochondrial metabolism in cells [6–8]. In recent years, studies have implicated cuproptosis is the occurrence and development of various cancers, including hepatocellular carcinoma, gastric cancer, and OSCC [9–12], suggesting that targeting cuproptosis could provide new and effective strategies for cancer treatment [6–12]. However, the regulatory mechanisms of cuproptosis have remained unclear, so the study of its regulatory mechanism is significant.

The biological clock of the human body is a chronoregulatory system that endogenously monitors and optimizes physiological activities [13] and has a crucial coordinative role in maintaining various aspects of physiological homeostasis, including body metabolism. The biological clock comprises several biological clock genes, abnormal alterations to which can lead to a variety of diseases such as cancer and metabolic diseases [14–18]. *Period2* (*PER2*) is a core biological clock gene [13, 16, 17], and downregulation of its expression promotes the development of a variety of cancers, including OSCC, lung cancer, and breast cancer [19–21]. Recently, *PER2* has also been found to be important in regulating cellular mitochondrial metabolic functions [22–24]. *PER2* and cuproptosis are both related to cellular mitochondrial metabolism, we hypothesized that *PER2* could have an important regulatory role in cuproptosis.

Starting from the novel perspective that the biological clock regulates cuproptosis, in the present study, and explored the role of the ATF3-PER2-AKT pathway in regulating cuproptosis in OSCC cells. For the first time, we explored upstream and downstream regulation of *PER2* in OSCC, and found that low expression of *PER2* promoted OSCC development by inhibiting cuproptosis; moreover, activating transcription factor 3 (ATF3) was the key upstream transcription factor that regulated *PER2* expression. This led to the discovery of a previously unknown mechanism in OSCC cells, by which overexpression of *ATF3* transcriptionally upregulates *PER2*, increases PER2 and HSP70 binding, and decreases HSP70 and AKT interaction, resulting in a decrease in the stability of AKT, which in turn promotes cuproptosis by inhibiting the AKT pathway. In addition, we demonstrated that the anti-tumor effect of ATF3 inducer 1 was significantly enhanced compared with monotherapy when it was combined with induction of cuproptosis by copper ionophore elesclomol (ES) in an OSCC xenograft model. Collectively, the results of this study demonstrate the critical role of *PER2* in regulating cuproptosis in OSCC cells, as well as identifying the upstream mechanisms that regulate *PER2* expression. The results also provide a basis for ATF3-targeted upregulation of *PER2* combined with induction of cuproptosis as a novel approach to potentially enhance the effectiveness of OSCC treatment.

## Results

### Low expression of *PER2* in OSCC and positive correlation with cuproptosis

**Fig. 1:**
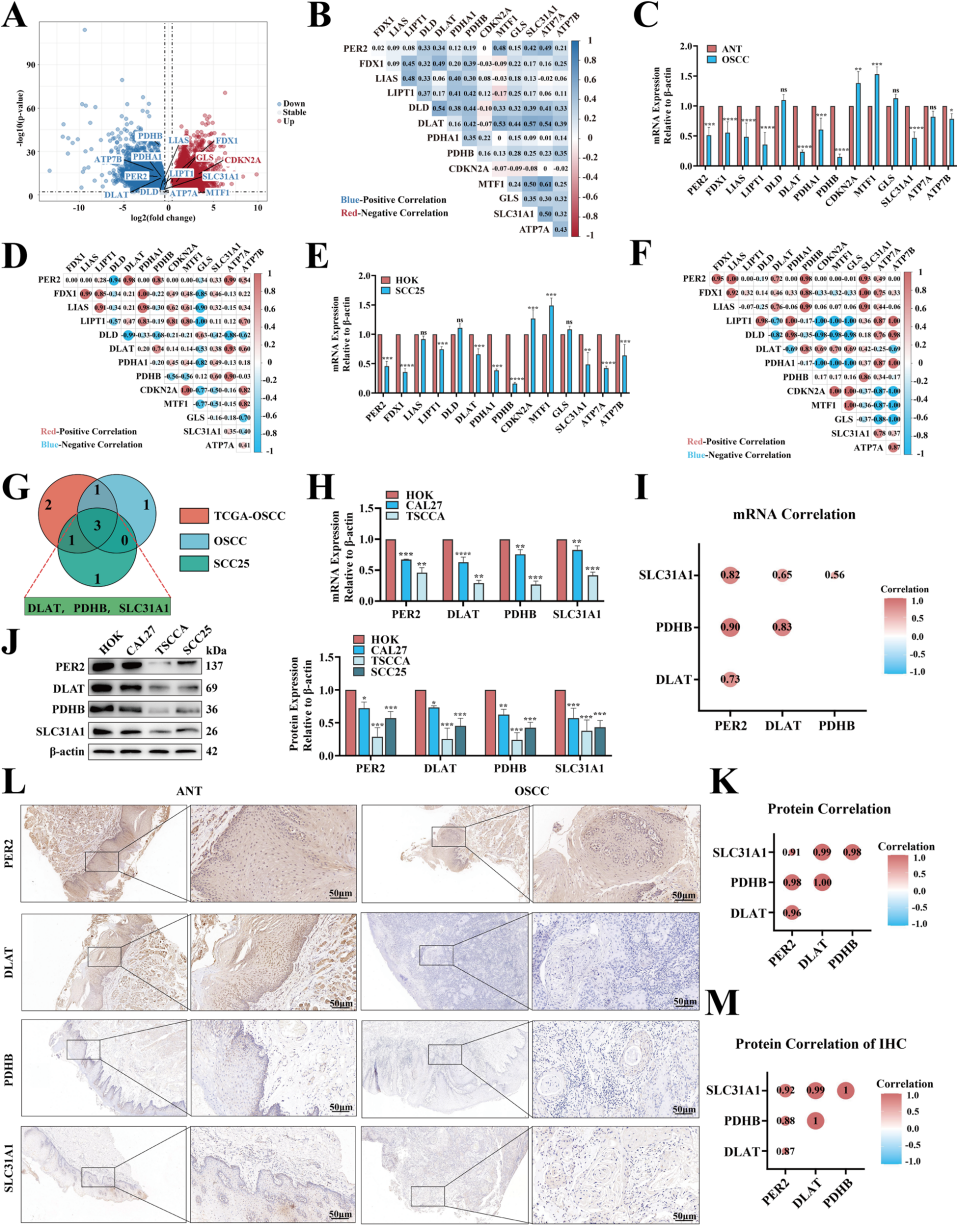
Low expression of PER2 in OSCC and positive correlation with cuproptosis. TCGA database and Pearson correlation analysis of the differences (**A**) and correlation (**B**) of the expression of *PER2* and 13 cuproptosis genes in OSCC compared with normal tissues. RT-qPCR assay and Pearson correlation analysis of 26 pairs of human OSCC and paired adjacent normal tissues for differences in expression (**C**) and correlation (**D**) of *PER2* and 13 cuproptosis genes. RT-qPCR assay and Pearson correlation analysis of the differences (**E**) and correlation (**F**) of the expression of *PER2* and 13 cuproptosis genes in SCC25 cells compared with HOK cells. **G** Venn diagrams take intersection results (correlation factor threshold >0.15). RT-qPCR detection of differential expression of *PER2* and 3 cuproptosis genes, *DLAT*, *PDHB*, and *SLC31A1* mRNA, in CAL27 and TSCCA cells (**H**) and correlation analysis by Pearson’s method (**I**). Western blotting to detect the expression of PER2, DLAT, PDHB, and SLC31A1 proteins in CAL27, TSCCA, and SCC25 cells (**J**) and Pearson correlation analysis (**K**). IHC assay of PER2, DLAT, PDHB, and SLC31A1 protein expression in OSCC tissues (*n* = 26, scale bars = 50 μm) (**L**) and Pearson correlation analysis (**M**). All data represent three replicate independent experiments. Data are presented as mean ± SD. *p < 0.05; **p < 0.01; ***p < 0.001; ****p < 0.0001. ANT adjacent normal tissue.

In previous study, we found that low *PER2* expression was associated with significantly shorter survival time in OSCC patients [19]. Here, to explore the relationship between *PER2* and cuproptosis in OSCC, we analyzed the correlations of *PER2* expression levels with those of 13 cuproptosis genes (*FDX1*, *LIAS*, *LIPT1*, *DLAT*, *DLD*, *PDHA1*, *PDHB*, *CDKN2A*, *MTF1*, *GLS*, *SLC31A1*, *ATP7A*, and *ATP7B*), which have been identified by genome-wide CRISPR/Cas9 loss-of-function studies and other techniques [6, 8]. To investigate the relation between *PER2* and cuproptosis, we analyzed the expression differentials and correlations between *PER2* and 13 cuproptosis genes in OSCC by three methods. First, data from TCGA were utilized for analysis (Fig. 1A, B and Table S1). Second, human OSCC tissue samples and paired adjacent normal tissues were determined by Reverse Transcription quantitative Polymerase Chain Reaction (RT-qPCR) (Fig. 1C, D). Third, human OSCC (SCC25) cells were examined by RT-qPCR (Fig. 1E, F). Finally, three cuproptosis-related genes (*DLAT*, *PDHB*, and *SLC31A1*) were selected for further investigation by taking the intersection of the results obtained using each of the above three methods in a Venn diagram, using significant expression differences and correlations as filtering criteria. The mRNA expression of these three cuproptosis genes was significantly downregulated in the TCGA-OSCC data and in OSCC tissues and SCC25 cells, and it was significantly positively correlated with *PER2* mRNA expression (Fig. 1G). By RT-qPCR, we also detected significant downregulation of *DLAT*, *PDHB*, and *SLC31A1* mRNA expression in two other OSCC cell types (CAL27 and TSCCA); again, the mRNA expression levels of these genes were significantly positively correlated with *PER2* mRNA expression (Fig. 1H, I). We further validated these results at the protein level. Western blotting demonstrated that the expression of DLAT, PDHB and SLC31A1 proteins was significantly reduced in the three OSCC cell types compared with HOK cells, and that it was significantly positively correlated with PER2 protein expression (Fig. 1J, K). Similarly, IHC showed a significantly reduced expression of PER2, DLAT, PDHB and SLC31A1 in OSCC tissues compared with adjacent normal tissues, and PER2 expression was significantly positively correlated with the expression of DLAT, PDHB, and SLC31A1 (Fig. 1L, M). DLAT and PDHB are key enzymes in the mitochondrial TCA cycle metabolic pathway, and SLC31A1 is a copper transporter protein that transports copper into the cell; their low expression thus results in inhibition of cuproptosis. Therefore, these results indicate that *PER2* may regulate cuproptosis in OSCC.

### *PER2*-dependent promotion of cuproptosis inhibits OSCC cell proliferation in vitro

**Fig. 2:**
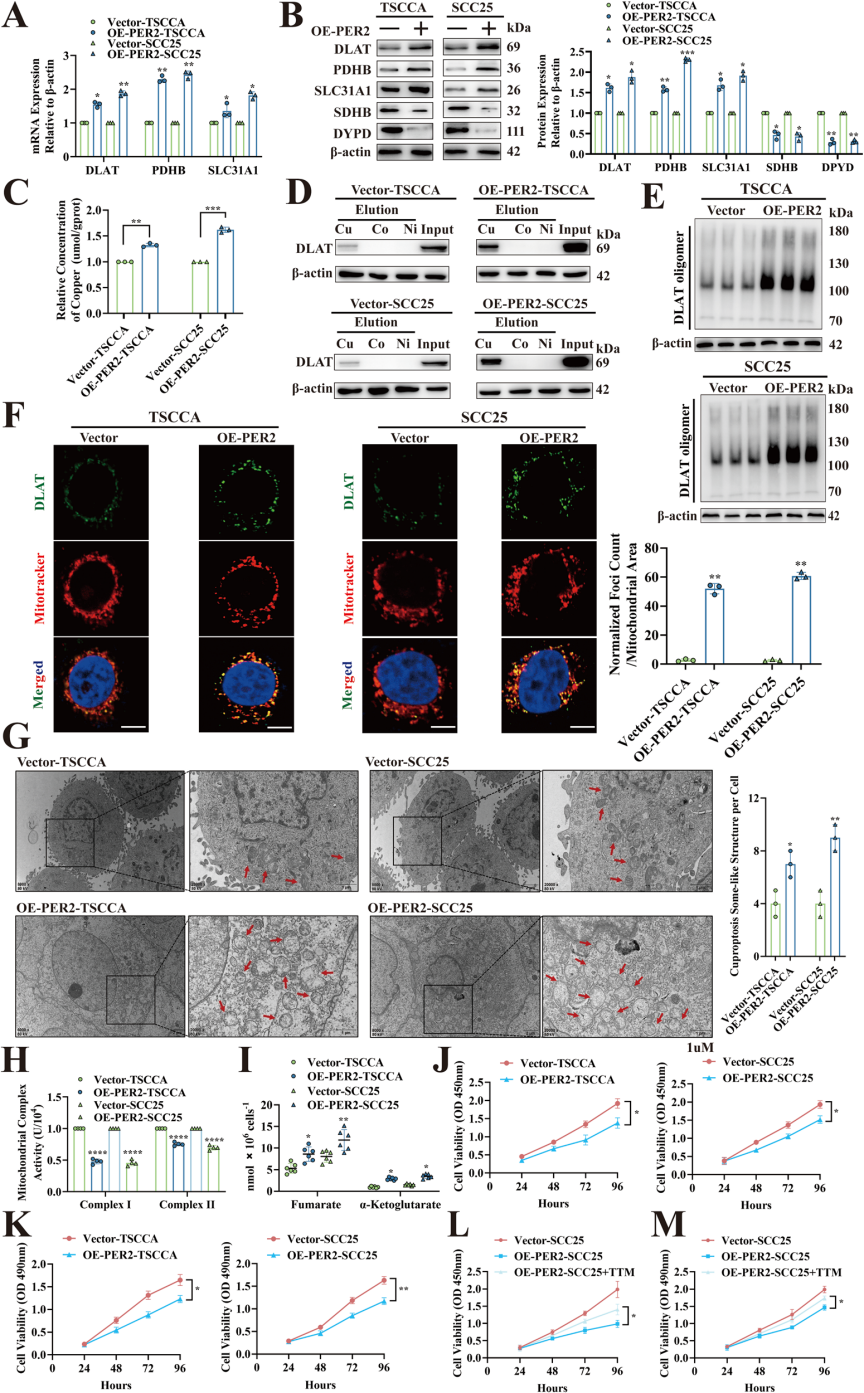
*PER2*-dependent promotion of cuproptosis inhibits OSCC cell proliferation in vitro. **A** RT-qPCR to determine *DLAT*, *PDHB* and *SLC31A1* mRNA expression in OSCC cells with overexpression of *PER2*. **B** Western blotting to determine DLAT, PDHB, SLC31A1, SDHB and DYPD protein expression in OSCC cells with overexpression of *PER2*. **C** Copper Colorimetric Assay Kit to determine the concentration of copper in OSCC cells with overexpression of *PER2*. **D** Copper affinity chromatography assay to determine the binding of copper to DLAT protein in OSCC cells with overexpression of *PER2*. **E** Non-denaturing gel electrophoresis assay to detect DLAT oligomers in OSCC cells with overexpression of *PER2*. **F** Immunofluorescence assay to detect DLAT oligomers in OSCC cells with overexpression of *PER2* (yellow, DLAT oligomer; green, DLAT; red, Mitotracker; blue, DAPI; scale bars = 50 μm; three independent experiments). **G** TEM to observe and quantify the number of vacuolated mitochondria in OSCC cells with overexpression of *PER2*, as well as the observation that mitochondria appeared to be deformed and swollen (red arrows indicate mitochondria; three independent experiments). **H** Micro-mitochondrial Complex I and II Activity Assay Kit to detect activity of mitochondrial complexes I and II in OSCC cells with overexpression of *PER2*. **I** Fumarate Assay Kit and α-KG Assay Kit to detect the concentration of TCA intermediate metabolites (fumarate and α-ketoglutarate) in OSCC cells with overexpression of *PER2*. **J** CCK-8 assay to determine levels of cell proliferation in OSCC cells with overexpression of *PER2*. **K** MTT assay to determine levels of cell proliferation in OSCC cells with overexpression of *PER2*. **L** CCK-8 assay showed a significant increase in proliferation level of OE-PER2*-*SCC25 cells after the addition of copper chelator TTM compared with OE-PER2-SCC25 cells. **M** MTT assay showed a significant increase in proliferation level of OE-PER2-SCC25 cells after the addition of copper chelator TTM compared with OE-PER2-SCC25 cells. All data represent three replicate independent experiments. Data are presented as mean ± SD. *p < 0.05; **p < 0.01; ***p < 0.001; ****p < 0.0001.

To identify the effect of *PER2* in the regulation of cuproptosis in OSCC cells, two *PER2*-overexpressing OSCC cell types (Overexpression of *PER2*: OE-*PER2*, OE-PER2-TSCCA and OE-PER2-SCC25) were established, and three *PER2*-silenced OSCC cell strains with stable knockdown were generated using three distinct short hairpin RNA (shRNA) sequences (sh-PER2-CAL27#1, sh-PER2-CAL27#2 and sh-PER2-CAL27#3) (Fig. S1A, B). RT-qPCR and western blotting showed significant increases in mRNA and protein expression for DLAT, PDHB, and SLC31A1 and significant decreases for iron-sulfur cluster proteins SDHB and DPYD in OE-PER2-TSCCA and OE-PER2-SCC25 cells (Fig. 2A, B). Intracellular copper and copper affinity binding assays showed that the intracellular copper concentrations of OE-PER2-TSCCA and OE-PER2-SCC25 cells were significantly increased (Fig. 2C), and copper binding to DLAT was significantly enhanced (Fig. 2D). Non-denaturing gel electrophoresis and immunofluorescence assays showed that levels of DLAT oligomers were significantly increased in OE-PER2-TSCCA and OE-PER2-SCC25 cells (Fig. 2E, F). Transmission electron microscopy and metabolite assays showed significant increases in numbers of vacuolated mitochondria (Fig. 2G), significant decreases in activity of mitochondrial electron transport chain complexes I and II (Fig. 2H), and significant increases in levels of intermediate metabolites of the TCA cycle (fumarate and α-ketoglutarate) in both OE-PER2-TSCCA and OE-PER2-SCC25 cells (Fig. 2I). CCK-8 and MTT assays also demonstrated a significantly reduced proliferation of OE-PER2-TSCCA and OE-PER2-SCC25 cells (Fig. 2J, K). However, in CAL27 cells with *PER2* silencing through three specific targets, the opposite results were obtained (Fig. S1C–J). Furthermore, when sh-*PER2*#3 lentivirus was transferred into OE-PER2-SCC25 cells to silence *PER2* for replication validation, the above results were significantly rescued (Fig. S2A–J). To further explore whether PER2 inhibition of OSCC cell proliferation was dependent on cuproptosis, we added tetrathiomolybdate (TTM), a copper chelator that reduces intracellular copper concentration to inhibit cuproptosis to OE-PER2-SCC25 cells for replication validation. The reduction in proliferation of OE-PER2-SCC25 cells was significantly reversed by the addition of TTM (Fig. 2L, M). These results suggest that *PER2* inhibits OSCC cell proliferation in vitro by promoting cuproptosis.

### *PER2*-dependent promotion of cuproptosis inhibits OSCC development in vivo

**Fig. 3:**
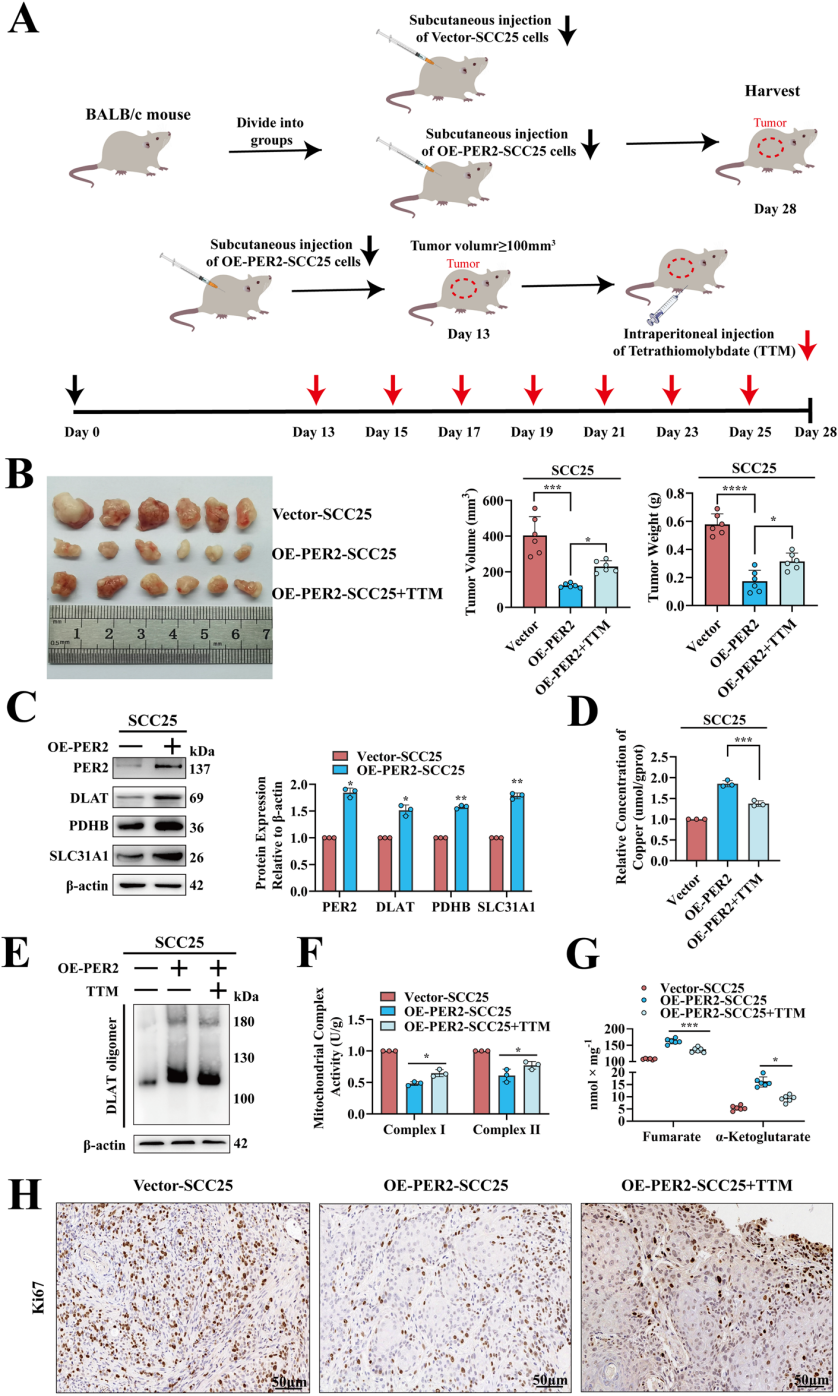
*PER2*-dependent promotion of cuproptosis inhibits OSCC development in vivo. **A** Schematic diagram of male BALB/c nude mice subcutaneously injected with Vector-SCC25 or OE-PER2-SCC25 cells to establish a model, and treated with TTM in OE-PER2-SCC25 model. **B** OE-PER2-SCC25 cell subcutaneous tumor formation assay in nude mice, growth of tumors in three groups at day 28. **C** Western blotting to examine PER2, DLAT, PDHB and SLC31A1 protein expression in tumors of Vector-SCC25 and OE-PER2*-*SCC25 groups. **D** Copper Colorimetric Assay Kit to measure intra-tumor copper concentration in Vector-SCC25, OE-PER2-SCC25 and OE-PER2-SCC25 + TTM groups. **E** Non-denaturing gel electrophoresis assay to detect intra-tumor DLAT oligomers in Vector-SCC25, OE-PER2-SCC25 and OE-PER2-SCC25 + TTM groups. **F** Micro-mitochondrial Complex I and II Activity Assay Kit to detect mitochondrial complex I and II activity in the tumors of Vector-SCC25, OE-PER2-SCC25 and OE-PER2-SCC25 + TTM groups. **G** Fumarate Assay Kit and α-KG Assay Kit to detect concentrations of fumarate and α-ketoglutarate in tumors of Vector-SCC25, OE-PER2-SCC25, and OE-PER2-SCC25 + TTM groups. **H** IHC assay of Ki67 expression in tumors in Vector-SCC25, OE-PER2-SCC25, and OE-PER2-SCC25 + TTM groups (*n* = 6, scale bars = 50 μm). All data represent three replicate independent experiments. Data are presented as mean ± SD. *p < 0.05; **p < 0.01; ***p < 0.001; ****p < 0.0001.

To evaluate the effects of PER2 in vivo, we established a subcutaneous tumorigenic model using OE-PER2-SCC25 cells in nude mice (Fig. 3A). Compared with those in the Vector-SCC25 group, tumors in the OE-PER2-SCC25 group had a significantly reduced weight and volume (Fig. 3B); a significantly increased expression of PER2, DLAT, PDHB, and SLC31A1 (Fig. 3C); significantly increased intra-tumor concentrations of copper and DLAT oligomers (Fig. 3D, E); a significantly decreased electron transport chain complex I and II activity (Fig. 3F); significantly increased fumarate and α-ketoglutarate levels (Fig. 3G); and a significantly decreased Ki67 expression (Fig. 3H). However, these changes in tumor weight, volume, and cuproptosis-related effects were markedly reversed in the OE-PER2-SCC25 + TTM group (Fig. 3B–H). These results indicate that *PER2* inhibits OSCC cell proliferation and tumor growth in vivo by promoting cuproptosis.

### *PER2* regulates OSCC cuproptosis through PER2/HSP70/AKT complex formation and AKT pathway

**Fig. 4:**
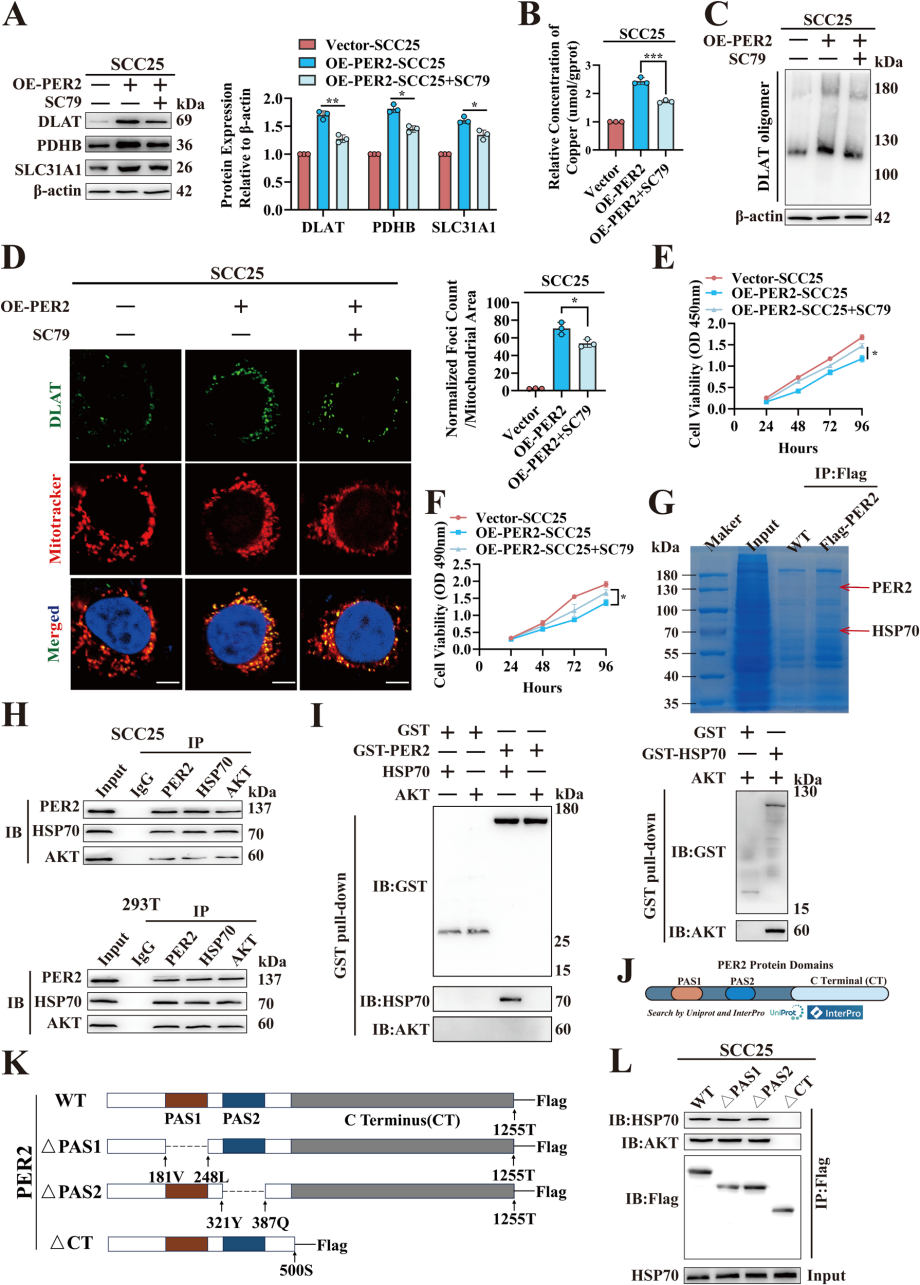
*PER2* regulates OSCC cuproptosis through PER2/HSP70/AKT complex formation and AKT pathway. **A** Western blotting showed that DLAT, PDHB, and SLC31A1 protein expression was significantly reduced by the addition of SC79 to OE-PER2-SCC25 cells. **B** Copper Colorimetric Assay Kit assay showed a significant reduction in copper concentration with the addition of SC79 to OE-PER2-SCC25 cells. **C** Non-denaturing gel electrophoresis assay detected a significant reduction of DLAT oligomers after addition of SC79 to OE-PER2-SCC25 cells. **D** Immunofluorescence assay detected a significant reduction in DLAT oligomers after addition of SC79 to OE-*PER2*-SCC25 cells (yellow, DLAT oligomer; green, DLAT; red, Mitotracker; blue, DAPI; scale bars = 50 μm; three independent experiments). **E** CCK-8 assay showed, cell proliferation levels were significantly increased by the addition of SC79 to OE-PER2-SCC25 cells. **F** MTT assay showed, cell proliferation levels were significantly increased by the addition of SC79 to OE-PER2-SCC25 cells. **G** Thomas blue-stained gel showed, Flag antibody fishing for candidate proteins that may bind to PER2 in SCC25 cells transfected with Flag-PER2. Schematic shows that three subunits (HSPA1, HSPA4 and HSPA8) of HSP70 in immunoprecipitation mass spectrometry results are all candidate proteins for binding to PER2 (screening criterion is unique peptide ≥2), suggesting that PER2 may have strong binding to HSP70. **H** Co-IP assay for PER2, HSP70 and AKT binding in SCC25 and 293 T cells. **I** GST pull-down assay for detecting direct binding of PER2 to HSP70 and AKT in vitro and direct binding of HSP70 to AKT in vitro. **J** Schematic showed that the major structural domains in UniProt and InterPro databases where PER2 binds to protein interactions are PAS1, PAS2, and C-terminal structural domain. **K** Construction of three plasmids with Flag-tagged *PER2* deletion mutations in the structural domains of PAS1 (region 181V-248L), PAS2 (region 321Y-387Q) and CT (region 500S-1255T), respectively. **L** Co-IP assays were performed to detect the binding of HSP70 and AKT after three deletion mutant plasmids transfection into SCC25 cells, respectively. All data represent three replicate independent experiments. Data are presented as mean ± SD. *p < 0.05; **p < 0.01; ***p < 0.001; ****p < 0.0001.

In previous study, we found that under-expression of *PER2* activated the AKT pathway, thereby promoting OSCC growth [20]. Recently Li et al. reported that inhibiting the AKT pathway promotes cuproptosis in hepatocellular carcinoma [9]. We therefore hypothesized that *PER2* would regulate cuproptosis in OSCC through the AKT pathway. To test this hypothesis, we added AKT activator SC79 (HY-18749, MCE) to OE-*PER2*-SCC25 cells. Addition of SC79 resulted in significant reversion of the effects of PER2 overexpression on DLAT, PDHB and SLC31A1 expression; intracellular copper concentration; levels of DLAT oligomers; activity of mitochondrial electron transport chain complexes I and II; fumarate and α-ketoglutarate levels; and level of cell proliferation (Figs. 4A–F and S3A, B). These results suggest that *PER2* promotes OSCC cuproptosis by inhibiting the AKT pathway.

We then further explored the mechanism underlying these effects. PER2 belongs to the PAS structural domain family and exerts its regulatory function primarily through protein-protein binding [25]. We therefore performed immunoprecipitation mass spectrometry of SCC25 cells after transfection with the Flag-*PER2* plasmid; HSP70 was found to be an abundant protein in the Flag-IP (Fig. 4G and Table S2), suggesting that HSP70 is a candidate protein for binding to PER2. As HSP70 has previously been observed to enhance AKT stability by binding to AKT in colorectal cancer cells [26], we hypothesized that a PER2/HSP70/AKT complex would form in OSCC cells, with effects on AKT stability. Co-immunoprecipitation (Co-IP) results demonstrated that in SCC25 and 293 T cells, PER2, HSP70, and AKT indeed formed a complex (Fig. 4H), and the GST pull-down assay indicated that PER2 interacted directly with HSP70 to form the PER2/HSP70 complex but could not bind directly to AKT (Fig. 4I). To further examine the structural regions of PER2 interaction with HSP70, we initially performed protein-protein docking prediction, which showed that PER2 binds to HSP70 protein mainly through its C-terminal structural domain (Fig. S3C). Then, we queried the UniProt (https://www.uniprot.org/) and InterPro databases (http://www.ebi.ac.uk/interpro/) and found that the primary structural regions of PER2 that bound to the interaction of protein were the PAS1, PAS2, and C-terminal structural regions (Fig. 4J). Therefore, we deleted the PAS1, PAS2, and C-terminal structural regions to produce three separate Flag-tagged *PER2* deletion mutants (Flag-Mut-PER2^ΔPAS1^-SCC25, Flag-Mut-PER2^ΔPAS2^-SCC25 and Flag-Mut-PER2^ΔCT^-SCC25) (Fig. 4K). The PER2/HSP70/AKT complex was found in Flag-Mut-PER2^ΔPAS1^-SCC25 and Flag-Mut-PER2^ΔPAS2^-SCC25 cells but, not in Flag-Mut-PER2^ΔCT^-SCC25 cells (Fig. 4L), consistent with the results of the Co-IP assay. These results indicate that the C-terminal structural region of PER2 interacts with HSP70, resulting in the formation of a PER2/HSP70/AKT complex.

### *PER2* promotes OSCC cuproptosis by downregulating HSP70 binding to AKT

**Fig. 5:**
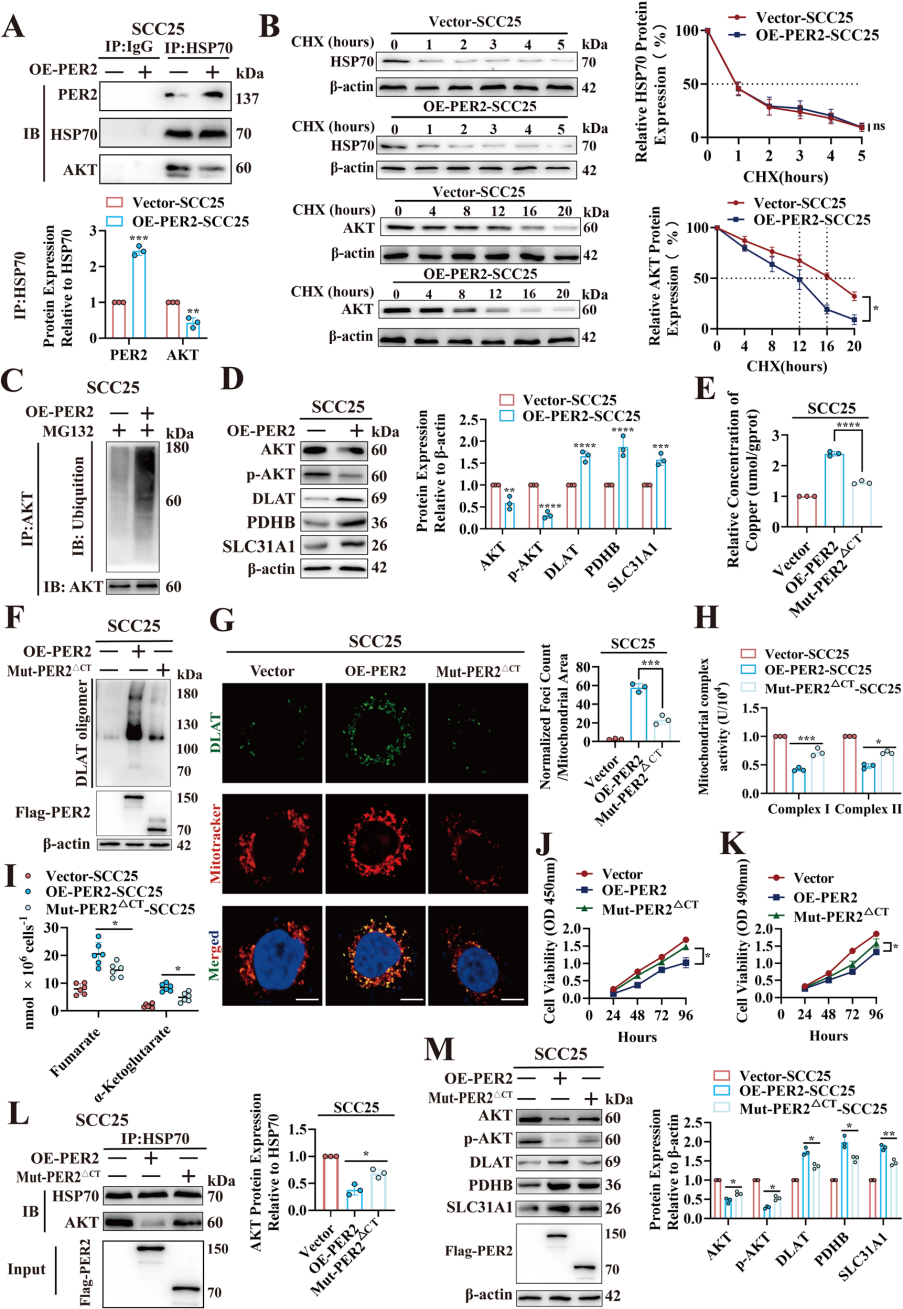
*PER2* promotes OSCC cuproptosis by downregulating HSP70 binding to AKT. **A** Co-IP assay for PER2/HSP70 and HSP70/AKT complexes in Vector-SCC25 and OE-PER2-SCC25 cells. **B** CHX chase assay for half-life of HSP70 and AKT in Vector-SCC25 and OE-PER2-SCC25 cells. **C** Western blot and strip assay for levels of AKT ubiquitination in Vector-SCC25 and OE-PER2-SCC25 cells. **D** Western blotting assays for AKT, p-AKT, DLAT, PDHB and SLC31A1 protein expression in Vector-SCC25 and OE-PER2-SCC25 cells. **E** Copper Colorimetric Assay Kit for detecting copper concentration in Vector-SCC25, OE-PER2-SCC25 and Mut-PER2^ΔCT^-SCC25 cells. **F** Non-denaturing gel electrophoresis assay for detection of DLAT oligomers in Vector-SCC25, OE-PER2-SCC25 and Mut-PER2^ΔCT^-SCC25 cells. **G** Immunofluorescence assay for DLAT oligomers in Vector-SCC25, OE-PER2-SCC25 and Mut-PER2^ΔCT^-SCC25 cells (yellow, DLAT oligomer; green, DLAT; red, Mitotracker; blue, DAPI; scale bars = 50 μm; three independent experiments). **H** Micro-mitochondrial Complex I and II Activity Assay Kit to measure activity of mitochondrial complexes I and II in Vector-SCC25, OE-PER2-SCC25, and Mut-PER2^ΔCT^-SCC25 cells. **I** Fumarate Assay Kit and α-KG Assay Kit to examine fumarate and α-ketoglutarate concentrations in Vector-SCC25, OE-PER2-SCC25, and Mut-PER2^ΔCT^-SCC25 cells. **J** CCK-8 assay for proliferation levels of Vector-SCC25, OE-PER2-SCC25 and Mut-PER2^ΔCT^-SCC25 cells. **K** MTT assay for proliferation levels of Vector-SCC25, OE-PER2-SCC25 and Mut-PER2^ΔCT^-SCC25 cells. **L** Co-IP showed a significant increase in HSP70/AKT complex in Mut-PER2^ΔCT^-SCC25 cells compared with OE-PER2-SCC25 cells. **M** Western blotting demonstrated significant increases in AKT and p-AKT and significant decreases in DLAT, PDHB and SLC31A1 protein expression in Mut-PER2^ΔCT^-SCC25 cells compared with OE-PER2-SCC25 cells. All data represent three replicate independent experiments. Data are presented as mean ± SD. *p < 0.05; **p < 0.01; ***p < 0.001; ****p < 0.0001.

The effect of the PER2/HSP70/AKT complex on the AKT pathway was further investigated. We hypothesized that the binding of PER2 to HSP70 would reduce binding of HSP70 and AKT, thereby inhibiting the AKT pathway by destabilizing AKT. To test this hypothesis, we performed a Co-IP assay; the results showed significantly increased levels of the PER2/HSP70 complex and significantly decreased levels of the HSP70/AKT complex in OE-PER2-SCC25 cells compared with Vector-SCC25 cells (Fig. 5A), suggesting that increasing PER2 binding to HSP70 leads to decreased HSP70 binding to AKT. We also performed a CHX chase assay, the results of which showed no significant difference in the half-life of HSP70 protein in OE-PER2-SCC25 cells, whereas that of AKT protein was significantly decreased (Fig. 5B), indicating a decrease in AKT stability. We further investigated the causes of decreased AKT stability; western blot and strip assay results showed a significant increase in ubiquitination levels of AKT in OE-PER2-SCC25 cells (Fig. 5C), suggesting that reduced formation of the HSP70/AKT complex led to increased intracellular AKT ubiquitination degradation. Western blotting results also showed that AKT and p-AKT levels were markedly decreased in OE-PER2-SCC25 cells, accompanied by significant increases in expression of cuproptosis-associated proteins DLAT, PDHB, and SLC31A1 (Fig. 5D); significant increases in intracellular concentrations of copper and DLAT oligomers (Fig. 5E–G); significant decreases in activity of mitochondrial electron transport chain complexes I and II (Fig. 5H); a significant accumulation of fumarate and α-ketoglutarate (Fig. 5I); and significantly reduced levels of cell proliferation (Fig. 5J, K). Furthermore, we validated the C-terminal structural domain reversion by mutating *PER2* and showed that the above effects on cuproptosis, the HSP70/AKT complex, and AKT, p-AKT, DLAT, PDHB, and SLC31A1 protein expression were all significantly rescued in Flag-Mut-PER2^ΔCT^-SCC25 cells (Fig. 5E–M). These results indicate that PER2 decreases the binding of HSP70 to AKT after binding to HSP70 through its C-terminal structural region, resulting in ubiquitinated degradation of AKT, thereby promoting cuproptosis and inhibiting proliferation of OSCC cells by suppressing the AKT pathway.

### *PER2* promoter binds to transcription factor ATF3 to activate *PER2* transcription

**Fig. 6:**
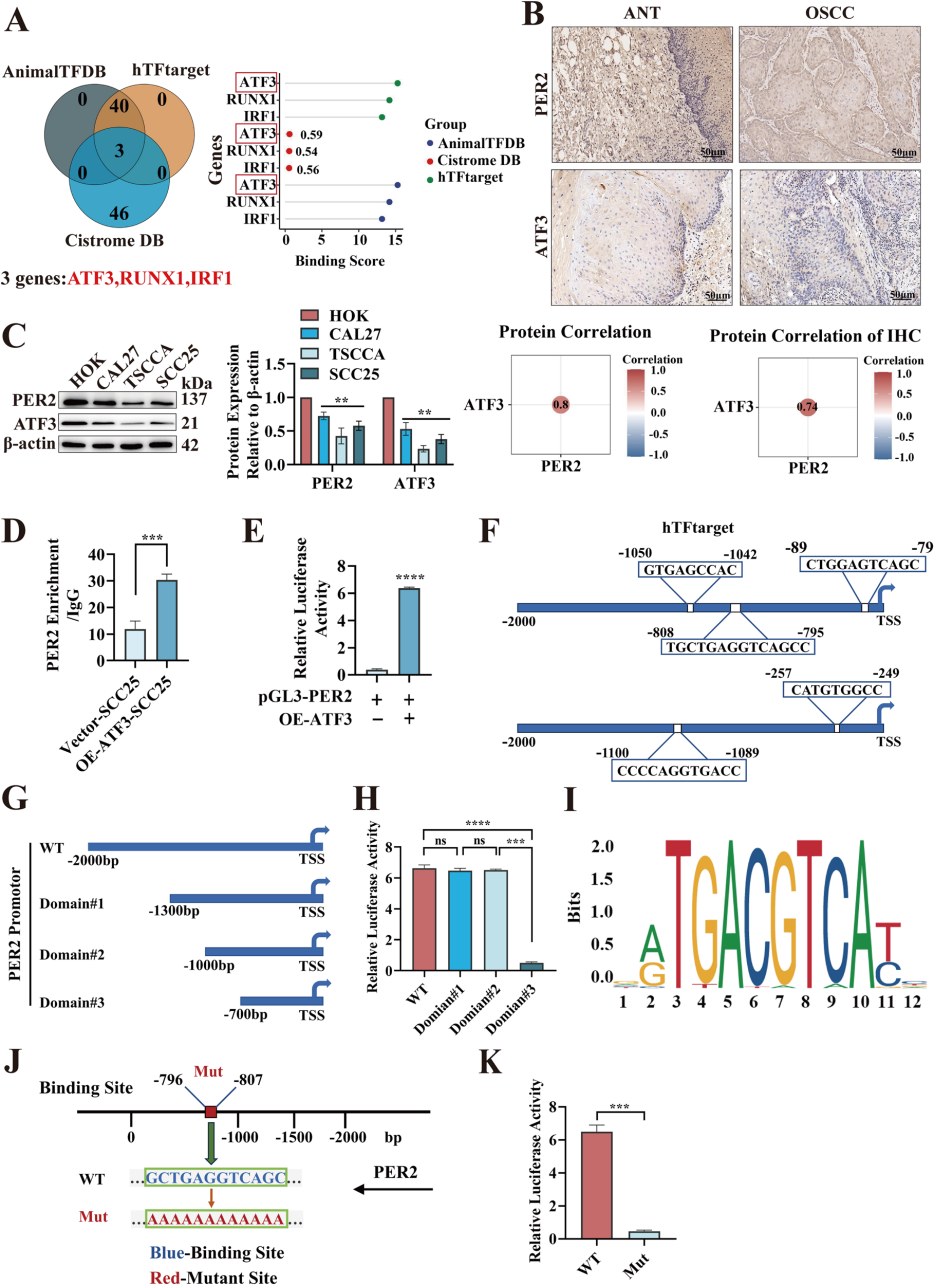
*PER2* promoter binds to transcription factor ATF3 to activate *PER2* transcription. **A** Venn diagram showed intersection of transcription factors regulating *PER2* from three databases, with binding rankings in order of ATF3, RUNX1, and IRF1. The *PER2* promoter starts 2000 bp before the transcription start site. **B** IHC assay of PER2 and ATF3 expression in OSCC tissues and Pearson correlations between PER2 and ATF3 protein expression (*n* = 26, scale bars = 50 μm, ANT adjacent normal tissue). **C** Western blotting of PER2 and ATF3 protein expression in CAL27, TSCCA and SCC25 cells, and Pearson correlations between expression of PER2 and ATF3 proteins. **D** ChIP assay showed significantly enhanced binding of ATF3 to the *PER2* promoter in OE-ATF3-SCC25 cells compared with Vector-SCC25 cells. **E** Dual-luciferase reporter gene assay to detect regulation of the *PER2* promoter by OE-ATF3. **F** Analysis of hTFtarget database showed that ATF3 has five binding sites (−89 ~ −79, −257 ~ −249, −808 ~ −795, −1050 ~ −1042, and −1100 ~ −1089) with the PER2 promoter, and the TSS in the figure denotes the transcription start site. **G** Construction of truncated domain#1 (−1300 to 0), domain#2 (−1000 to 0), and domain#3 (−700 to 0) and wild-type (WT) *PER2* promoter nucleotide sequences. **H** Dual-luciferase reporter gene assay to detect regulation by ATF3 binding of the WT *PER2* promoter sequence and the three segment truncations. **I** Prediction based on the JASPAR database of the possible base sequence of ATF3 in the *PER2* promoter sequence (GCTGAGGTCAGC), and analysis of the conservation of each base position in this sequence. Each column corresponds to one base position, and each base position consists of a stack of bases at that position, and the greater the total height of the stack of bases (denoted as bits, i.e., the value of vertical coordinates), the greater the conservation of bases at that position. **J** Schematic representation of the putative ATF3 binding site in the *PER2* promoter and point mutation. Arrows indicate the direction of *PER2* sequence transcription direction; blue bases show the predicted binding site (i.e., the WT sequence GCTGAGGTCAGC); and red bases indicate the mutated (Mut) binding site (i.e., Mut sequence AAAAAAAAAAAA). **K** Dual-luciferase reporter gene assay to examine the role of ATF3 in regulating the nucleotide sequence of the *PER2* promoter in the WT and Mut groups. All data represent three replicate independent experiments. Data are presented as mean ± SD. *p < 0.05; **p < 0.01; ***p < 0.001; ****p < 0.0001.

Having found that *PER2* mRNA expression was decreased in OSCC, we explored the reason for the downregulation of *PER2* expression in OSCC at the transcriptional level. Transcription factors that target the *PER2* gene were downloaded from AnimalTFDB, hTFtarget and Cistrome DB (Table S3); after taking the intersection of the results from these three databases, we found that the top-ranked factor for binding to the *PER2* promoter was ATF3 (Fig. 6A). We thus examined the expression of ATF3 in OSCC. IHC results showed that ATF3 expression was markedly decreased in OSCC tissues and was significantly and positively related with expression of PER2 (Fig. 6B). Western blotting demonstrated that the expression of PER2 and ATF3 proteins was markedly downregulated in the three OSCC cell types compared with HOK cells, and ATF3 expression was again markedly positively correlated with PER2 expression (Fig. 6C).

Subsequently, we verified the binding of ATF3 to the *PER2* promoter. ChIP assay results showed that ATF3 interacted with the *PER2* promoter in SCC25 cells (Fig. 6D), and dual-luciferase reporter gene assay demonstrated that the luciferase activity of *PER2* was markedly enhanced in OE-ATF3-SCC25 cells compared with Vector-SCC25 cells (Fig. 6E), suggesting that ATF3 interacts with the *PER2* promoter to promote *PER2* transcription. Further analysis using the hTFtarget database revealed that ATF3 has five binding sites for binding to the *PER2* promoter (Fig. 6F); based on these, we constructed three truncations (Domain#1, Domain#2, and Domain#3) of the *PER2* promoter nucleotide sequence (Fig. 6G and Table S6) and transferred the truncated sequences into OE-ATF3-SCC25 cells. In the dual-luciferase reporter gene assay, strong fluorescence was observed in the wild-type, Domian#1 and Domian#2 groups, whereas no significant fluorescence was observed in the Domain#3 groups (Fig. 6H), suggesting that the ATF3 binding sites for the *PER2* promoter are in the range from −1000 to −700 bp. Subsequently, point mutations were introduced based on the binding sites of ATF3 to the *PER2* promoter obtained from the JASPAR database (Fig. 6I, J). In the dual-luciferase reporter gene assay, no significant fluorescence was detected in the Mut group (Fig. 6K). The above results indicate that ATF3 interacts with the *PER2* promoter to activate *PER2* transcription, and the binding site is located in the region of −807 to −796 bp.

### ATF3 promotes OSCC cuproptosis dependent on PER2

**Fig. 7:**
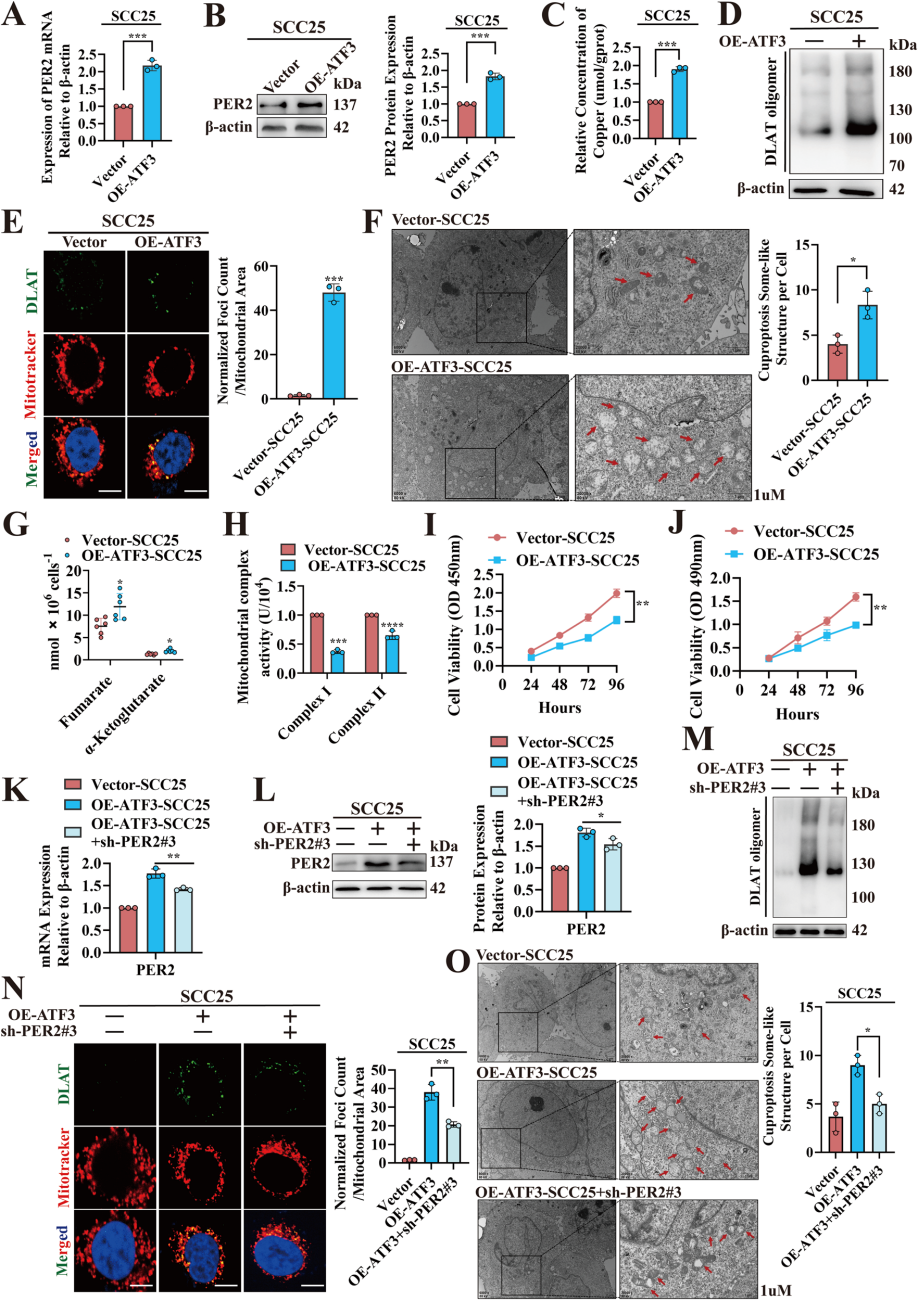
ATF3 promotes OSCC cuproptosis dependent on PER2. **A** RT-qPCR to detect *PER2* mRNA expression in Vector-SCC25 and OE-ATF3-SCC25 cells. **B** Western blotting to detect PER2 protein expression in Vector-SCC25 and OE-ATF3-SCC25 cells. **C** Copper Colorimetric Assay Kit for measuring copper concentration in Vector-SCC25 and OE-ATF3-SCC25 cells. **D** Non-denaturing gel electrophoresis assay for analyzing DLAT oligomers in Vector-SCC25 and OE-ATF3-SCC25 cells. **E** Immunofluorescence assay for DLAT oligomers in Vector-SCC25 and OE-ATF3-SCC25 cells (yellow, DLAT oligomer; green, DLAT; red, Mitotracker; blue, DAPI; scale bars = 50 μm; three independent experiments). **F** TEM to observe the number of vacuolated mitochondria in Vector-SCC25 and OE-ATF3-SCC25 cells (red arrows indicate mitochondria; three independent experiments). **G** Fumarate Assay Kit and α-KG Assay Kit to detect fumarate and α-ketoglutarate concentrations in Vector-SCC25 and OE-ATF3-SCC25 cells. **H** Micro-mitochondrial Complex I and II Activity Assay Kit to detect activity of mitochondrial complexes I and II in Vector-SCC25 and OE-ATF3-SCC25 cells. **I** CCK-8 assay for proliferation levels of Vector-SCC25 and OE-ATF3-SCC25 cells. **J** MTT assay for proliferation levels of Vector-SCC25 and OE-ATF3-SCC25 cells. **K** RT-qPCR assay showed significantly lower mRNA expression of *PER2* in OE-ATF3-SCC25+sh-*PER2*#3 cells compared with OE-ATF3-SCC25 cells. **L** Western blotting showed a marked reduction in PER2 protein expression in OE-ATF3-SCC25+sh-*PER2*#3 cells compared with OE-ATF3-SCC25 cells. **M** Non-denaturing gel electrophoresis assay to examine significant decreases in DLAT oligomers in OE-ATF3-SCC25+sh-*PER2*#3 cells compared with OE-ATF3-SCC25 cells. **N** Immunofluorescence assay showed significant decreases of DLAT oligomers in OE-ATF3-SCC25+sh-*PER2*#3 cells compared with OE-ATF3-SCC25 cells. **O** TEM observed significant decreases in the number of vacuolated mitochondria, as well as decreases in mitochondrial deformation and swelling in OE-ATF3-SCC25+sh-*PER2*#3 cells compared with OE-ATF3-SCC25 cells (red arrows denote mitochondria, three independent experiments). All data represent three replicate independent experiments. Data are presented as mean ± SD. *p < 0.05; **p < 0.01; ***p < 0.001; ****p < 0.0001.

To investigate the effect of ATF3 in regulation of *PER2* and cuproptosis, we constructed OSCC cells (OE-ATF3-SCC25) stably overexpressing *ATF3* (Fig. S4A, B). These cells showed significantly increased PER2 mRNA and protein expression, intracellular copper concentration, levels of DLAT oligomers, numbers of vacuolated mitochondria, and levels of fumarate and α-ketoglutaric (Fig. 7A−G), and significantly decreased mitochondrial electron transport chain complex I and II activity and cell proliferation (Fig. 7H−J). Next, we transfected sh-*PER2*#3 lentivirus into OE-ATF3-SCC25 cells for replication validation; the above cuproptosis-related effects were significantly reversed in these OE-ATF3-SCC25 + sh-*PER2*#3 cells (Figs. 7K−O and S4C−F). Further in vivo validation was performed by a subcutaneous tumor formation assay in nude mice (Fig. S4G). Compared with those of the vector-SCC25 group, the masses and sizes of tumors in the OE-ATF3-SCC25 group were significantly decreased (Fig. S4H), whereas concentrations of copper and DLAT oligomers in tumors were significantly increased (Fig. S4I, J), and the expression of Ki67 was markedly reduced (Fig. S4K). These effects were significantly reversed in the OE-ATF3-SCC25 + sh-*PER2*#3 group (Fig. S4H–K). These in vivo and in vitro experimental results suggested that in OSCC, ATF3-dependent *PER2* promotes OSCC cuproptosis and inhibits cell proliferation.

Then, we verified the effects of ATF3 inducer 1 (HY-151923, MCE) on the regulation of *PER2* expression and cuproptosis. Addition of ATF3 inducer 1 to SCC25 cells resulted in significant increases in PER2 mRNA and protein levels (Fig. S5A, B), significant increases in intracellular copper concentration and DLAT oligomer levels (Fig. S5C–E), significant decreases in activity of mitochondrial electron transport chain complexes I and II (Fig. S5F), and marked increases in fumarate and α-ketoglutarate levels (Fig. S5G), whereas cell proliferation levels were significantly reduced (Fig. S5H, I). These results suggest that ATF3 inducer 1 can target and upregulate *PER2* to promote cuproptosis in OSCC.

### ATF3-targeted upregulation of *PER2* together with induction of cuproptosis improves efficacy in treatment of OSCC

**Fig. 8:**
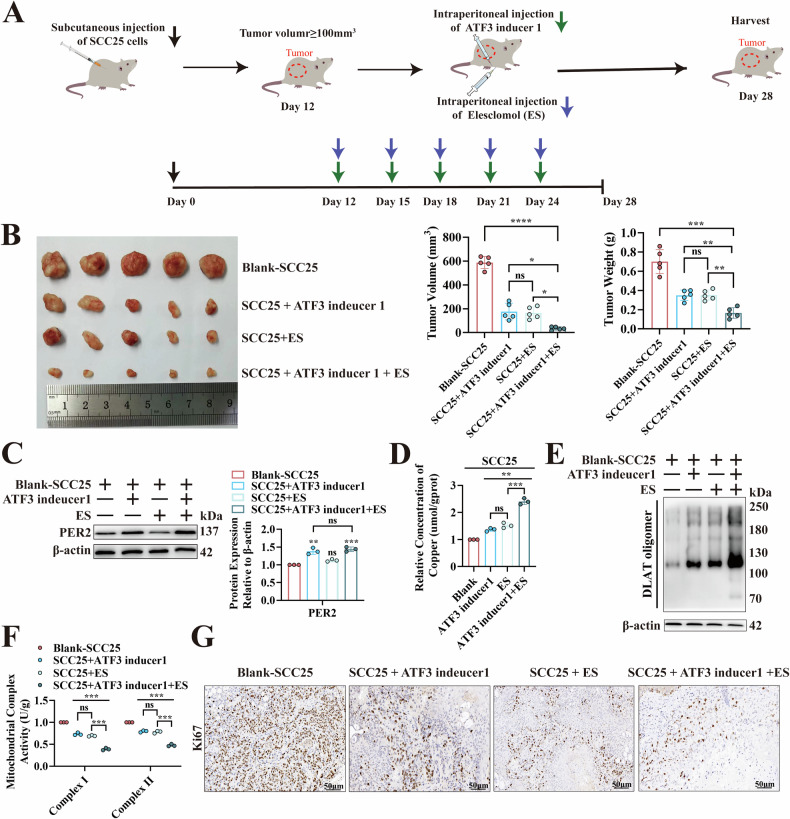
ATF3-targeted upregulation of *PER2* combined with induction of cuproptosis improves efficacy in OSCC treatment. **A** Schematic diagram of subcutaneous injection of SCC25 cells to establish an OSCC model in BALB/c nude mice, and anti-tumor treatment with ATF3 inducer 1 and ES. **B** Nude mice were injected subcutaneously with SCC25 cells, and after tumor formation, saline, ATF3 inducer 1, ES, and a combination of the two were injected according to Blank-SCC25, SCC25 + ATF3 inducer 1, SCC25 + ES, and SCC25 + ATF3 inducer 1 + ES treatment groups, respectively, and the weight and volume were measured after harvesting tumors on day 28. **C** Western blotting for PER2 protein expression in Blank-SCC25, SCC25 + ATF3 inducer 1, SCC25 + ES and SCC25 + ATF3 inducer 1 + ES groups. **D** Copper Colorimetric Assay Kit to measure intra-tumor copper concentrations in the Blank-SCC25, SCC25 + ATF3 inducer 1, SCC25 + ES and SCC25 + ATF3 inducer 1 + ES groups. **E** Non-denaturing gel electrophoresis assay to detect DLAT oligomers in Blank-SCC25, SCC25 + ATF3 inducer 1, SCC25 + ES and SCC25 + ATF3 inducer 1 + ES groups. **F** Micro-mitochondrial Complex I and II Activity Assay Kit for detecting activity of electron transport chain complexes I and II in Blank-SCC25, SCC25 + ATF3 inducer 1, SCC25 + ES and SCC25 + ATF3 inducer 1 + ES groups. **G** IHC assay of Ki67 expression in Blank-SCC25, SCC25 + ATF3 inducer 1, SCC25 + ES and SCC25 + ATF3 inducer 1 + ES groups (*n* = 5, scale bars = 50 μm). All data represent three replicate independent experiments. Data are presented as mean ± SD. *p < 0.05; **p < 0.01; ***p < 0.001; ****p < 0.0001.

We established a subcutaneous OSCC model using SCC25 cells in nude mice to explore the efficacy of ATF3 inducer 1 and copper ionophore ES for OSCC treatment (Fig. 8A). Compared with those of the Blank-SCC25 group, the masses and sizes of tumors were considerably lower in both the SCC25 + ATF3 inducer 1 and SCC25 + ES groups (Fig. 8B), whereas those in the SCC25 + ATF3 inducer 1 + ES combination treatment group were significantly lower compared with either of the single-treatment groups (Fig. 8B). ATF3 inducer 1 significantly upregulated PER2 expression in vivo (Fig. 8C). Compared with the Blank-SCC25 group, the SCC25 + ATF3 inducer 1, SCC25 + ES, and SCC25 + ATF3 inducer 1 + ES groups showed significantly increased intratumor copper concentration and DLAT oligomer levels (Fig. 8D, E), and significantly decreased activity of mitochondrial electron transport chain complexes I and II and Ki67 expression (Fig. 8F, G). These effects were significantly stronger in the SCC25 + ATF3 inducer 1 + ES group than in either single-treatment group (Fig. 8B–G). Thus, although either ATF3 inducer 1 or ES alone can promote cuproptosis to inhibit OSCC growth, their combination is significantly superior in terms of potential therapeutic efficacy.

## Discussion

Owing to the diversity and heterogeneity of cancers, the development of effective therapies to destroy cancer cells requires elucidation of the mechanisms underlying the occurrence of various forms of cell death [27]. In recent years, an unique form of cell death caused by copper, called cuproptosis, has been discovered [6]; it differs from previously known cell death mechanisms in that it involves the binding of copper to lipoylated proteins within mitochondrial TCA, which in turn leads to oligomerization of proteins, most commonly DLAT proteins within mitochondria, thus initiating cuproptosis [9, 28]. The main features of cuproptosis include significant increases in levels of intracellular copper, protein oligomers (e.g., DLAT oligomers), and TCA intermediary metabolites, and significant decreases in iron-sulfur cluster proteins, mitochondrial electron transport chain complex activity, and cellular proliferation, as well as mitochondrial morphologic abnormalities and dysfunction [6–8]. Increases in intracellular copper and DLAT oligomer levels represent the most important evidence for the occurrence of cuproptosis. Accumulating evidence from recent studies shows that cuproptosis is strongly correlated with the occurrence and progression of a variety of cancers [9–12], indicating potential new approaches to cancer treatment. However, the mechanisms underlying cuproptosis have not yet been fully elucidated; in particular, the upstream regulatory mechanism of cuproptosis is still poorly understood, with few studies having focused on this aspect. Li et al. reported that maternal embryonic leucine zipper kinase (MELK) inhibited cuproptosis through activation of PI3K/mTOR pathway, thereby promoting hepatocellular carcinoma development and progression [9]. Zhu et al. found that integrin β1 (ITGB1) promoted malignant progression of gastric cancer through inhibition of cuproptosis-related genes, including *FDX1* and *DLAT* [10]. Tian et al. reported that p32 promoted cuproptosis by interacting with cuproptosis-related protein DLAT, which in turn inhibited proliferation of renal cell carcinoma [29]. Here, we discovered a previously undescribed function of core biological clock gene *PER2* in mediating cellular cuproptosis, revealing that PER2 binding to HSP70 results in ubiquitination degradation of AKT, which in turn promotes the expression of DLAT, PDHB, and SLC31A1 and, consequently, promotes OSCC cuproptosis. The outcomes of this study contribute to our understanding of the mechanisms regulating cuproptosis.

Low expression of *PER2* has been recognized as an independent prognostic variable in several cancers, including OSCC [19, 21]; however, the reasons for the reduced expression of *PER2* in these cancers have not been fully determined. Further exploration of this topic will be important for the design of combination therapies based on targeting of *PER2*. In this study, we demonstrated through bioinformatics analysis and a variety of in vitro and in vivo assays that ATF3 expression is decreased in OSCC, that ATF3 interacts with the *PER2* promoter to activate *PER2* transcription, and that the promotion of cuproptosis by ATF3 in OSCC is dependent on *PER2*. ATF3 is a member of the ATF/cAMP response element binding family and has crucial functions in regulation of metabolic homeostasis of organisms and the development of various cancers [30–33]. For example, Xu et al. reported that low expression of ATF3 could promote the development of tongue squamous cell carcinoma [32]. The results of the present study not only extend our understanding of the mechanism by which *ATF3* promotes tumorigenesis but also provide new insights into the upstream regulatory mechanism of *PER2*. Moreover, treatment of subcutaneous OSCC in nude mice with either ATF3 inducer 1 (which targets upregulation of *PER2*) or ES (a copper ionophore that induces cuproptosis) resulted in significant efficacy, but the strategy combining the two treatments showed significantly improved efficacy compared with either single treatment. These results indicate that such a combination may represent a novel approach for the treatment of OSCC.

Cuproptosis provides a new direction for cancer treatment, and recent studies have also found that cuproptosis engages in cross-talk with regulatory pathways including tumor immunity, chemoresistance, and ferroptosis [12, 34, 35]. These findings may provide further insight into combined treatment strategies involving cuproptosis. The present study demonstrated that overexpression of *ATF3* or *PER2* promotes cuproptosis in OSCC cells by upregulating expression of cuproptosis-related genes *DLAT*, *PDHB*, and *SLC31A1*. Other recent findings have shown that *DLAT* has a pivotal role in cuproptosis, moreover, regulates PD-L1 expression and enhances the infiltration of tumor-associated macrophages and regulatory T cells [35–38], suggesting a synergistic effect between cuproptosis and tumor immunotherapy. Increased expression of SLC31A1, a copper transporter protein that transports copper into the cell, promotes cuproptosis [35]; SLC31A1 also functions as a transporter protein for platinum anticancer drugs such as cisplatin (CDDP), and its increased expression enhances cellular uptake of CDDP [39–41]. CDDP and immune checkpoint blockade (ICB) targeting the PD-1/PD-L1 pathway are first-line treatment protocols for the treatment of a range of malignancies, including OSCC [42–45]. However, tumor resistance to both CDDP and ICB represents a significant challenge that impedes their efficacy in the treatment of OSCC [46, 47]. The results of the present study suggest that combined strategies involving cuproptosis and CDDP or ICB could show significantly improved therapeutic efficacy in OSCC; this, thus is a line of research with potential clinical value.

In conclusion, this study has demonstrated that overexpression of *ATF3* promotes cuproptosis in OSCC cells via a mechanism dependent on *PER2*. Specifically, direct binding of ATF3 to the *PER2* promoter transcriptionally upregulates *PER2*, which decreases the interaction of HSP70 with AKT by increasing PER2 and HSP70 binding, leading to ubiquitinated degradation of AKT and thus promoting OSCC cuproptosis by inhibiting the AKT pathway. A combination protocol involving cuproptosis, induced by ATF3 inducer 1-targeted upregulation of PER2 and ES, demonstrated efficacy in a mouse subcutaneous OSCC model, indicating a highly advantageous novel approach to enhance the efficacy of OSCC treatment.

## Materials and methods

### Clinical samples

Human OSCC tissues and paired adjacent normal tissues (at least 5 mm distant from the tumor) were obtained from inpatients with OSCC undergoing oral and maxillofacial surgery at the First Hospital of Chongqing Medical University. All participants had their diagnosis confirmed by pathology analysis, and no participants underwent any preoperative treatment such as radiotherapy and chemotherapy. All procedures of the study were conducted in accordance with the Declaration of Helsinki, written informed consent was obtained from all patients, and the study was approved by the Ethics Committee of First Affiliated Hospital of Chongqing Medical University (approval number: 2021-588).

### Cell culture

Shanghai Huiying Biotechnology Co., Ltd. supplied normal human oral keratin cells (HOK cells). CAL27 and SCC25 cells were supplied by Starfish Biotechnology Co., Ltd. Shanghai Zhongqiao Xinzhou Biotechnology Co., Ltd. supplied the TSCCA cells. All cell lines were identified using short tandem repeat analysis, and it was subsequently confirmed that none of the cell lines had any mycoplasma contamination. The cell culture medium consisted of 10% fetal bovine serum (S711-001S, Uruguay), 1% penicillin-streptomycin, and 89% Dulbecco’s modified Eagle medium (Gibco, USA). Cells were grown in an incubator with 5% CO_2_ at 37 °C.

### Vector construction

Lentiviruses were designed and synthesized by GeneChem (Shanghai, China). The three target short hairpin RNA (shRNA) sequences presented in Table S4 were used to construct *PER2*-silencing lentiviruses (sh-*PER2*#1, sh-*PER2*#2, and sh-*PER2*#3). A lentivirus overexpressing *PER2* (OE-*PER2*) was also obtained.

The *PER2* deletion mutant plasmid and lentiviral vector were devised and manufactured by Hanheng Biologicals (Shanghai, China) Co. A Flag-tagged *PER2* plasmid (Flag-*PER2*) and *PER2* deletion mutant plasmids (Flag-Mut-*PER2*^ΔPAS1^, Flag-Mut-*PER2*^ΔPAS2^, and Flag-Mut*-PER2*^ΔCT^) were obtained; their sequences are presented in Table S5.

The pGL3-*PER2* and OE-*ATF3* plasmids, OE-*ATF3* lentivirus and truncator were devised and manufactured by Lipovo Bio (Wuhan, China). A *PER2* full-length promoter reporter gene plasmid (pGL3-*PER2*) and ATF3 plasmid were synthesized, and primers for amplification of the three *PER2* promoter truncations, Domain#1 (0–1300 bp), Domain#2 (0–1000 bp), and Domain#3 (0–700 bp), were devised; the primer sequences are presented in Table S6. A *PER2* promoter truncation plasmid was obtained. *PER2* promoters with point mutations in the 796–807 bp range were designed and synthesized by Lipovo Bio; the primer sequences are presented in Table S7. All the plasmids were subjected to DNA sequencing for verification.

### RT-qPCR assay

Total RNA was extracted using a Super FastPure Cell RNA Isolation Kit (RC102-01, Vazyme), following the manufacturer’s instructions. With Oligo V.7.0 software, PCR primers were generated for *PER2*, *FDX1*, *LIAS*, *LIPT1*, *DLAT*, *DLD*, *PDHA1*, *PDHB*, *MTF1*, *GLS*, *CDKN2A*, *SLC31A1*, *ATP7A*, *ATP7B*, and *β-actin*; primer sequences are presented in Table S8. The 2^−∆∆Ct^ approach was utilized to determine the relative mRNA expression of the target genes, with *β-actin* serving as a reference.

### Western blotting

RIPA lysis buffer (P0013B, Beyotime, China) containing 1% phosphatase inhibitor and 1% phenylmethylsulfonyl fluoride solution was used to obtain cell or tissue lysates. A BCA Protein Quantification Kit (P0013B, Beyotime, China) was used to measure protein concentrations. Protein samples (30 μg) were separated by 8–12.5% sodium dodecyl sulfate polyacrylamide gel electrophoresis (SDS-PAGE) and transferred onto polyvinylidene difluoride (PVDF) membranes (Merck Millipore). The PVDF membranes were immersed in 5% milk and blocked for 1.5 h. Following blocking, primary antibodies bound to the membranes overnight at 4 °C. The primary antibodies against PER2, DLAT, PDHB, SLC31A1, SDHB, DPYD, HSP70, AKT, p-AKT, Flag, ubiquitin (P4D1), ATF3, and β-actin are presented in Table S9. The membranes were then incubated with horseradish peroxidase-labeled secondary antibodies at 37 °C for 1 h. β-actin was utilized as a control, and the matching total proteins were employed as controls for phosphorylated proteins. Protein band gray values were measured with ImageJ 5.0 software.

### Immunohistochemistry (IHC)

IHC assays were performed following the manufacturer’s instructions with an SP-9000 IHC Assay Kit (ZSGB-BIO, China); information on the antibodies used is given in Table S9. Results were analyzed following a two-score semi-quantitative method.

### Copper assay

Tumor and intracellular copper concentrations were quantified with a Copper Colorimetric Assay Kit (E-BC-K300-M and E-BC-K775-M, Elabscience, China) and calculated according to the following formula: copper concentration (μmol/gprot) = (ΔA580 − b) ÷ a ÷ Cpr × f.

### Copper affinity binding assay

Profinity^TM^ IMAC Resin (Biorad #1560121, Bio-Rad) was washed and loaded with metal (0.2 M CoCl_2_, NiCl_2_, or CuCl_2_) following the manufacturer’s instructions. Cells were lysed with RIPA buffer and loaded onto the column. The column was then washed three times with 1×PBS and eluted with 300 mM imidazole (elution buffer). Western blotting was performed on the eluted proteins.

### Non-denaturing gel electrophoresis assay

Cell or tissue homogenates were lysed with NP40 buffer (P0013F, Beyotime, China), and the protein concentrations of the samples were quantified with a BCA Protein Quantification Kit. Native-PAGE was run at 90 V for 1 h at no load. Then, 30 µg of each protein sample was separated by native-PAGE and transferred to a PVDF membrane for 3.5 h. The samples were blocked in 5% milk for 1.5 h at room temperature, and then incubated successively with DLAT antibody and β-actin secondary antibody. Finally, an ultra-sensitive ECL Chemiluminescence Kit (BeyoECL Plus, P0018S, Beyotime) was used to examine the proteins.

### Immunofluorescence assay

Cells were placed in confocal Petri dishes, left over-night, and processed as indicated. For mitochondrial staining, in a 37 °C cell culture incubator, 500 nM of MitoTracker® Red CMXRos (1:2000, M7512, Thermo Fisher) was added to the cells for 30 min. They were then fixed with 4% paraformaldehyde for 15 min, permeabilized with 0.1% Triton X-100 for 5 min, and blocked with 3% bovine serum albumin for 30 min, and incubated with DLAT antibody (1:100, T58125, Abmart) at 4 °C overnight. Then, cells were washed three times with 0.1% PBS-T (PBS containing 0.1% Tween-20), incubated with Alexa Fluor 488 anti-rabbit secondary antibody (1:1000, 4412S, CST) for 1 h at 37 °C, and stained with DAPI (C1005, Beyotime) for 10 min. Confocal microscopy (Dragonfly200, England) was used to capture images at ×600 magnification.

### DLAT foci segmentation and analysis

The DLAT foci segmentation method used here was similar to the procedure previously reported [6]. DLAT foci signals were analyzed with ImageJ software 1.54c. DLAT-stained images were first converted to 8-bit binary images based on an empirically determined threshold; foci areas below 40 pixels were identified as background signals and filtered out; and foci were then calculated after filtering out lesions below the threshold (40 pixels) or within the DAPI area. As there were significant differences in cell morphology and size between different experimental groups, foci counts were normalized to the mitochondrial area and expressed as foci count/mitochondrial area.

### Transmission electron microscopy (TEM)

After the samples had been processed, cells were digested and collected in centrifuge tubes and centrifuged at 1200 rpm for 10 min, and the supernatant was removed. A 4% glutaraldehyde fixation solution was slowly applied along the wall of the tube and left overnight at 4 °C. Then, cells were then embedded, sectioned, and stained, and finally observed and collected under a transmission electron microscope (Hitachi, H-75000, Japan).

### Mitochondrial electron transport chain complex I and II activity assay

Cell or tissue mitochondria were extracted using Mitochondrial Isolation Kits (C3601 and C3606, Beyotime, China) following the manufacturer’s instructions and preserved in mitochondrial storage solution (C3609, Beyotime) for subsequent experiments. Mitochondrial complex I and II activities were measured using CheKine^TM^ Micro-mitochondrial Complex I and II Activity Assay Kits (KTB1850 and KTB1860, Abbkine, China) following the instructions provided. Mitochondrial complex I and II activities were calculated using the formula ΔA = A_1_ − A_2_, where A_1_ is the initial absorbance value at 0 min and A_2_ is the absorbance value at 2 min at 340 nm and 605 nm, respectively.

### Fumarate and α-ketoglutarate assay

Quantitative analysis of fumarate and α-ketoglutarate was performed using a Fumarate Assay Kit (MAK060, Sigma, USA) and α-KG Assay Kit (ab83431, Abcam) according to the instructions provided. The absorbance values of fumarate and α-ketoglutaric assay were read at 450 nm and 570 nm, respectively, and converted using standard curves.

### CCK-8 assay

The assay was performed following the instructions of the CCK-8 Kit (BS350B, Biosharp, China). Briefly, three wells per group of a 96-well plate were each filled with 100 μL of cell suspension at a density of 1 × 10^4^ cells. The optical density (OD) was measured at 24, 48, 72, and 96 h. Cell proliferation was assessed by plotting a cell growth curve with time as the horizontal axis and the OD value as the vertical axis.

### MTT assay

The assay was performed using an MTT Kit (BL132B, Biosharp, China) following the manufacturer’s instructions. Briefly, cell suspensions with a density of 1 × 10^4^ cells/mL were added to 96-well plates (100 μL per well), with three wells for each group. The OD was measured at 24, 48, 72, and 96 h, and cell proliferation was assessed by plotting the cell growth curve with time as the horizontal axis and the OD value as the vertical axis.

### Immunoprecipitation mass spectrometry

The Flag-PER2 plasmid was transfected into SCC25 cells, and samples were collected after 48 h. Extracts were immunoprecipitated to obtain proteins interacting with PER2. The proteins were then separated using, 10% SDS-PAGE, and stained with Caulmers Brilliant Blue. Excised gel strips were cut into three pieces of approximately 1 mm. The gels were then subjected to protein in-gel digestion. The peptides obtained were separated and identified using a TRIPLE TOF 6600 mass spectrometer, and the resulting mass spectrometry data were imported into Maxquant 2.4.2.0 software (Cox and Mann, Germany) for analysis.

### Protein–protein docking prediction

To determine the specific structural domains of HSP70 that bound to PER2, we obtained structure files for human-derived HS71A protein (AF-P0DMV8-F1) and human-derived PER2 protein (AF-O15055-F1) from the AlphaFold database (https://alphafold.ebi.ac.uk/). The structures were submitted to the ClusPro server (https://cluspro.org/) for rigid-body docking, and docked conformations were selected for analysis according to coefficient weight calculation, combining the output cluster distribution results and the weight scoring ranking. The results were visualized with PyMOL 2.4.0 (Schrödinger, USA), and protein interactions were analyzed using Ligplot^+^ v.2.2.4 (EMBL-EBI, Europe).

### Co-immunoprecipitation (Co-IP)

Flag, IgG, PER2, HSP70, or AKT antibody (1 µg, at a dilution of 1:100) was added to cell lysates; see Table S9 for antibody information. The lysates were then incubated at 4 °C overnight and immunoprecipitated using Protein A/G magnetic beads. The coprecipitated samples were assayed by western blotting.

### GST pull-down assay

Plasmids containing the glutathione-S-transferase (GST) PER2 or GST-HSP70 sequence were introduced into *Escherichia coli*, and isopropyl β-d-thiogalactopyranoside (IPTG) was used to stimulate protein expression. Then, GST pull-down analysis was conducted using a Pierce GST Protein Interaction Pull-down Kit (21516, Thermo Fisher, USA), following the manufacturer’s instructions. Glutathione agarose was added to bacterial lysates, followed by incubation for 1 h at 4 °C to capture GST–PER2 and GST–HSP70 bridging proteins. After approximately 100 μg of fusion proteins and the target proteins had been incubated for 4 h in vitro, the bound proteins were eluted using glutathione elution buffer and subjected to western blotting.

### Western blot and strip assay

Levels of ubiquitinated AKT in the captured protein samples were determined by western blotting using AKT as the bait; antibody information is presented in Table S9. Protein band gray values were measured with ImageJ 5.0 software.

### Cycloheximide (CHX) chase assay

Cells were plated with 100 μg/mL CHX (Sigma-Aldrich, USA). Total HSP70 protein was obtained at 0 h, 1 h, 2 h, 3 h, 4 h, and 5 h, and total AKT protein was obtained at 0 h, 4 h, 8 h, 12 h, 16 h, and 20 h. The extracted protein samples were then detected by western blotting.

### Bioinformatics analysis

Differential expression and correlation analyses of *PER2* and 13 cuproptosis genes in OSCC were performed as follows. Transcriptome data of OSCC patients were downloaded from The Cancer Genome Atlas (TCGA-OSCC, downloaded on October 1, 2022, http://cancergenome.nih.gov/). After data extraction and transformation, the DESeq2 method was used for differential analysis of *PER2* and the 13 cuproptosis genes; *p* < 0.01 and |log2(fold change)| > 0.4 were the criteria used to identify differentially expressed genes. The Pearson method was used for correlation analysis of *PER2* and the 13 cuproptosis genes, with thresholds of correlation coefficient (R) > 0.15 and *p* < 0.001.

For the *PER2* transcription factor prediction analysis, transcription factors of *PER2* target genes were downloaded from AnimalTFDB (http://bioinfo.life.hust.edu.cn/AnimalTFDB#!/), hTFtarget (http://bioinfo.life.hust.edu.cn/hTFtarget/#!/), and Cistrome DB (http://cistrome.org/db/) on January 1, 2023. The three datasets were processed using the inclusion criteria “source: database”, “strand: +”, and “Q value ≤ 0.05”, and Venn diagrams were constructed to determine their intersection.

For prediction of *PER2* promoter site mutation binding site, the *PER2* promoter sequence was obtained from NCBI (https://www.ncbi.nlm.nih.gov/gene/), whereas the human ATF3 transcription factor was retrieved from the JASPAR database (https://jaspar.elixir.no/). Prediction was performed to compare ATF3 with the *PER2* promoter sequence using the JASPAR database (with a threshold of 80%), and the results showed the predicted binding site.

### Dual-luciferase reporter gene assay

The pGL3-PER2 reporter gene plasmid was cotransfected into SCC25 cells with the Renilla luciferase plasmid and ATF3 plasmid. Cotransfection of the vector plasmid and Renilla luciferase plasmid was used as a negative control. The expression was assayed for firefly luciferase activity (F) and Renilla luciferase activity (R), and relative luciferase activity was calculated as F/R.

### Chromatin immunoprecipitation (ChIP)

Expression of the *PER2* promoter was analyzed by RT-qPCR following the instructions of the ChIP Assay Kit (P2078, Beyotime, China), with the following primer sequences: forward: 5′-TATGTGGGAGAGCTACGCTG-3′, reverse: 5′-TCCTCCTCTTCGTGGCCTTA-3′. IgG was used as a control. *PER2* promoter amplification was performed for each group based on the CT value obtained by RT-qPCR.

### In vivo mouse experiments

Forty-seven male BALB/c nude mice (Strain NO.D000521, weight: 19–21 g, age: 5–6 weeks) were sourced from Gempharmatech Co., Ltd., China, and raised under specific-pathogen-free conditions in the Laboratory Animal Center of Chongqing Medical University. All animal experiments were approved by the Institutional Animal Care and Use committee of Chongqing Medical University (approval number: IACUC-CQMU-2024-0028).

For the experiments to investigate PER2-promoted inhibition of OSCC via cuproptosis, mice were blindly allocated at random into three groups (*n* = 6 per group) using the approach known as random number tables. In the Vector and OE-PER2-SCC25 groups, 0.2 mL samples of Vector-SCC25 and OE-PER2-SCC25 cell suspensions (concentration of 5 × 10^6^ cells/mL) were administered subcutaneously into the left backs of mice on day 0. In the OE-PER2 + TTM group, OE-PER2-SCC25 cell suspension was administered subcutaneously into the left backs of mice as above, and tetrathiomolybdate (TTM; 20 mg/kg, once for 2 days, a total of seven times) was given intraperitoneally on day 13 when the tumor volume was ≥100 mm^3^. On day 28, we dislocated the cervical vertebrae of the mice and extracted the tumors.

For the experiments to investigate ATF3-dependent PER2-promoting OSCC cuproptosis, mice were blindly allocated at random into three groups (*n* = 3 per group) using random number tables. Mice in the Vector group, OE-ATF3-SCC25 group, and OE-ATF3-SCC25 + sh-*PER2*#3 group received subcutaneous administration in the left back on day 0 of 0.2 mL of Vector-SCC25, OE-ATF3-SCC25, or OE-ATF3-SCC25 + sh-*PER2*#3 cell suspension, respectively (concentration of 5 × 10^6^ cells/mL). The formation of tumors was observed on day 9. On day 28, we dislocated the cervical vertebrae of the mice and extracted the tumors.

For tumor treatment experiments, on day 0, a suspension of SCC25 cells in PBS (0.2 mL, 5 × 10^6^ cells/mL) was allocated subcutaneously into the left backs of 20 nude mice, and the mice were blindly placed at random into four groups (*n* = 5 per group) using random number tables. Treatment was initiated on day 12 when the tumor volume was ≥100 mm^3^; the SCC25 group was used as the control group. Mice in the SCC25 + ATF3 inducer 1 group received intraperitoneal injection of ATF3 inducer 1 (40 mg/kg every 3 days, a total of 5 times). Mice in the SCC25 + ES group received intraperitoneal injection of ES (a copper ionophore that promotes cuproptosis; 10 mg/kg every 3 days, a total of 5 times). The SCC25 + ATF3 inducer 1 + ES combination treatment group received all the treatments, administered as above. On day 28, we dislocated the cervical vertebrae of the mice and extracted the tumors.

An electronic balance and a vernier caliper were used to quantify each tumor that was extracted in the above experiments; the tumor volume was obtained by the following formula: tumor volume = 0.5 × a × b^2^, where a and b are the maximum and minimum diameters of the tumor, respectively. The tumor tissues were employed in subsequent experimental assays.

### Statistical analysis

GraphPad Prism (v.9.5; GraphPad Software, La Jolla, USA) was used to handle and statistically analyze the data. The results obtained from three separate replicate experiments were expressed as mean ± SD. Correlation analysis was performed using the Pearson method. Comparisons between two independent sample groups were performed using Student’s *t* test, one-way comparisons among multiple groups were performed using one-way analysis of variance (ANOVA), and two-factor comparisons were performed using two-way ANOVA. A *p* value < 0.05 was deemed to indicate statistical significance.

## Supplementary information

Supplementary Figure S1-5

Supplementary Table S1

Supplementary Table S2

Supplementary Table S3

Supplementary Tables S4-9

Original western blot images

## Data Availability

The transcriptome data presented in this study are available in the Oral Squamous Carcinoma dataset of TCGA. Transcription factor and binding site data predicted in this study are available in four databases, AnimalTFDB, hTFtarget, Cistrome DB and JASPAR. All other data generated in this study are available upon request from the corresponding author. The proteomics data of this study are publicly available in iProx at IPX0009277000.
