# Supplementary material for: PER2 interaction with HSP70 promotes cuproptosis in oral squamous carcinoma cells by decreasing AKT stability
Source: Cell Death Dis. 2025 Mar 20;16(1):192. doi: 10.1038/s41419-025-07523-1 (PMC11926231; doi:10.1038/s41419-025-07523-1)
Supplement: Supplementary file 1 — Supplementary Figure S1-5 [file 41419_2025_7523_MOESM1_ESM.pdf]

Supplementary Figures 1-5 for

PER2 interaction with HSP70 promotes cuproptosis in oral squamous carcinoma cells by decreasing AKT stability

Wenguang Yu[1], Shilin Yin[1], Hong Tang[1], Hengyan Li[1], Zhiwei Zhang[1], and Kai Yang[1]

Figure S1

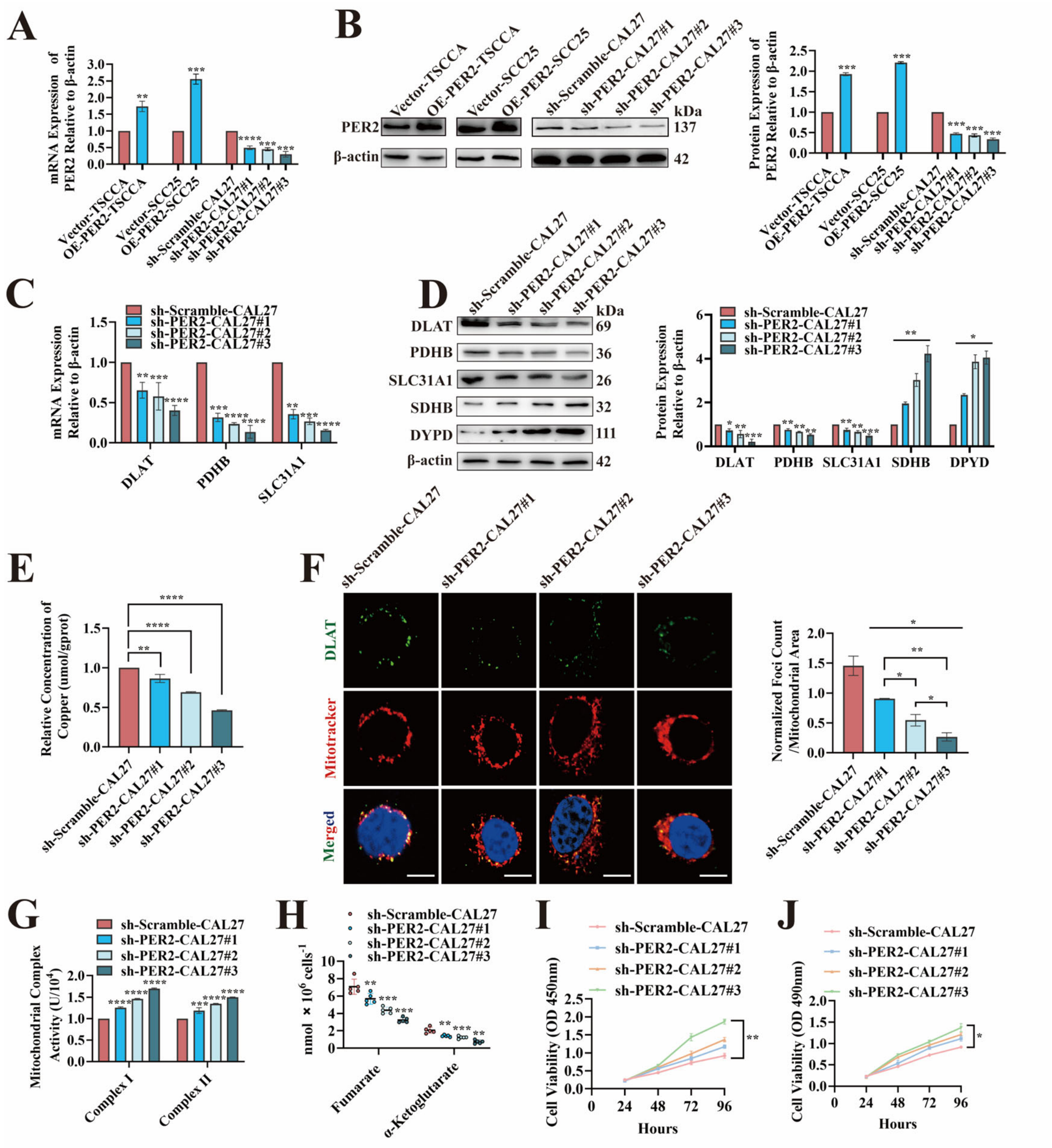

Figure S1. Construction of OSCC cells stably transfected with overexpressing or silencing *PER2* and inhibition of OSCC cuproptosis by silencing *PER2*

TSCCA and SCC25 cells with relatively low *PER2* expression were selected among three OSCC cell types to construct OE-PER2-TSCCA and OE-PER2-SCC25 cells stably overexpressing *PER2*, with *PER2* overexpression efficiencies of 1.93±0.03 and 2.21±0.02, respectively. Vector-TSCCA and Vector-SCC25 cells were used as control groups. CAL27 cells with relatively high *PER2* expression were selected, and sh-PER2-CAL27#1, sh-PER2-CAL27#2, and sh-PER2-CAL27#3

cells were constructed to stably silence *PER2* against three different effector targets, and sh-Scramble-CAL27 cells were used as a negative control. The PER2 silencing efficiencies were 52.9%±2.6%, 56.8%±4.0%, and 66.0%±3.3%, respectively. **A.** RT-qPCR showed significant increases in *PER2* mRNA expression in two OSCC cell types overexpressing *PER2* and significant decreases in *PER2* mRNA expression in OSCC cells silenced for *PER2* by three different effector targets, compared with control group. **B.** Western blotting showed significant increases in PER2 protein expression in two OSCC cell types overexpressing *PER2* and significant decreases in PER2 protein expression in OSCC cells from three different effector targets silencing *PER2*, as compared with control group. **C.** RT-qPCR showed that *DLAT*, *PDHB* and *SLC31A1* mRNA expression was significantly reduced in CAL27 cells with three different effector targets silencing *PER2* compared with control. **D.** Western blotting showed that DLAT, PDHB and SLC31A1 protein expression was significantly reduced in CAL27 cells with *PER2* silenced by three different effector targets compared with control. **E.** Copper Colorimetric Assay Kit detected significantly lower copper concentrations in CAL27 cells silenced with *PER2* at three different effector targets compared with control. **F.** Immunofluorescence assay detected significant reductions of DLAT oligomers in CAL27 cells with *PER2* silenced by three different effector targets compared with control (yellow, DLAT oligomer; green, DLAT; red, Mitotracker; blue, DAPI; scale bars = 50 μm; three independent experiments). **G.** Micro-mitochondrial Complex I and II Activity Assay Kit detected significant increases in electron transport chain complex I and II activity in CAL27 cells with *PER2* silenced by three different effector targets compared with control. **H.** Fumarate Assay Kit and α-KG Assay Kit showed that the concentrations of fumarate and α-ketoglutarate were significantly reduced in CAL27 cells with *PER2 s*ilenced by three different effector targets compared with control. **I.** CCK-8 assay showed that the proliferation levels of CAL27 cells with *PER2* silenced by three different effector targets were significantly decreased compared with control group. **J.** MTT assay showed that the proliferation levels of CAL27 cells with *PER2* silenced by three different effector targets were significantly increased compared with control group. All data represent three replicate independent experiments. Data are presented as mean ± SD. **P* < 0.05; ***P* < 0.01; ****P* < 0.001; *****P* < 0.0001.

Figure S2

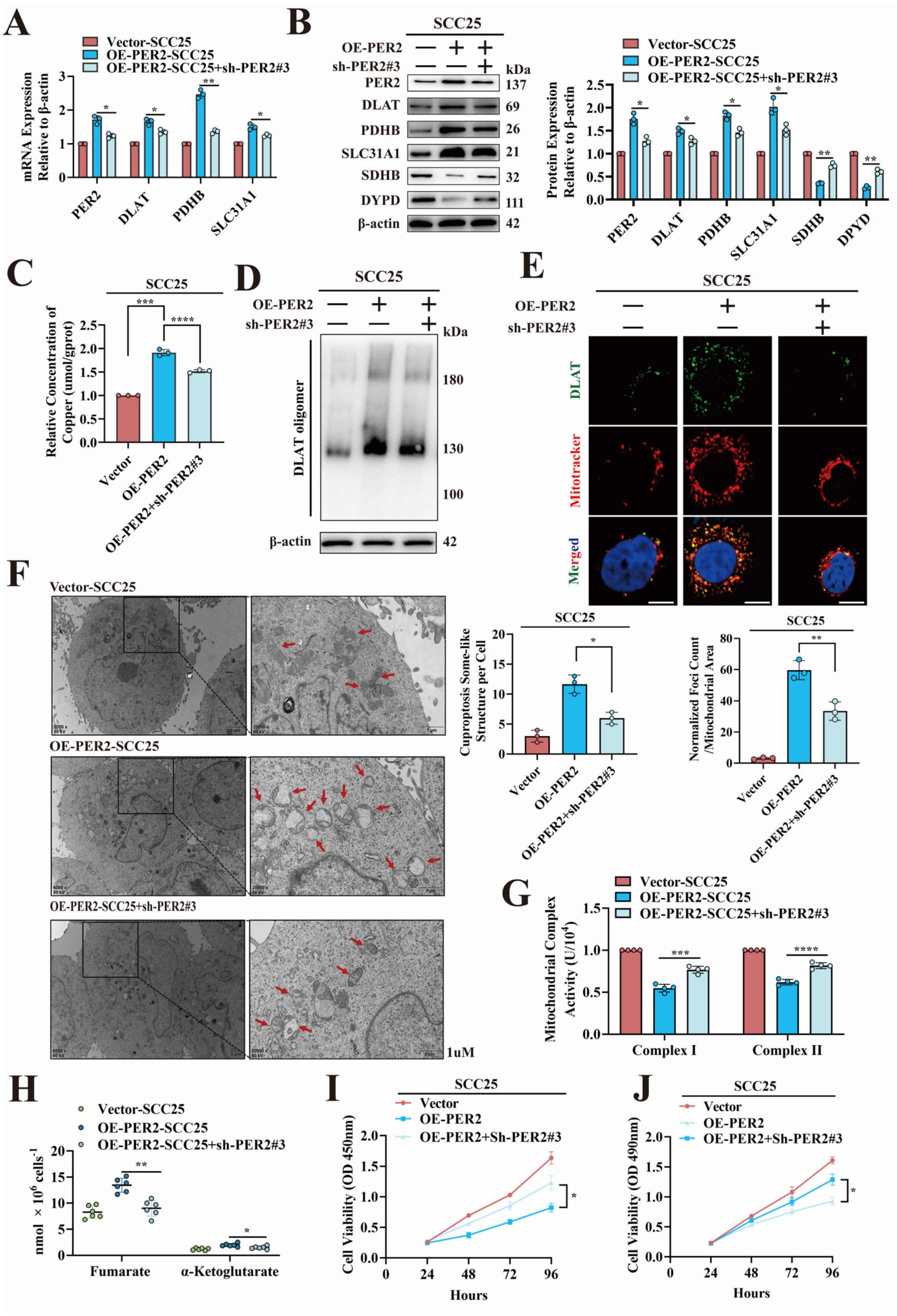

Figure S2. The occurrence of cuproptosis in OSCC is dependent on *PER2*

A. RT-qPCR showed, *PER2*, *DLAT, PDHB* and *SLC31A1* mRNA expression was significantly reduced in OE-PER2-SCC25 cells transfected with sh-*PER2*#3 as compared with OE-PER2-SCC25 cells. **B.** Western blotting showed, PER2, DLAT, PDHB, SLC31A1, SDHB, and DYPD protein expression was significantly reduced in OE-PER2-SCC25 cells transfected with sh-*PER2*#3 compared with OE-PER2-SCC25 cells. **C.** Copper Colorimetric Assay Kit showed a significant reduction of copper levels in OE-PER2-SCC25 cells transfected with sh-*PER2*#3 compared with OE-PER2-SCC25 cells. **D.** Non-denaturing gel electrophoresis assay showed the DLAT oligomers were reduced after transfection of OE-PER2-SCC25 cells with sh-*PER2*#3 compared with OE-PER2-SCC25 cells. **E.** Immunofluorescence assay showed, DLAT oligomers were significantly reduced in OE-PER2-SCC25 cells transfected with sh-*PER2*#3 compared with OE-PER2-SCC25 cells (yellow, DLAT oligomer; green, DLAT; red, Mitotracker; blue, DAPI; scale bars = 50 μm; three independent experiments). **F.** TEM observed a significant decrease in the amount of vacuolated mitochondria, as well as a reduction in mitochondrial deformation and swelling, after transfecting sh-*PER2*#3 into OE-PER2-SCC25 cells as compared with OE-PER2-SCC25 cells (red arrows indicate mitochondria; three independent experiments). **H.** Fumarate Assay Kit and α-KG Assay Kit detected concentrations of fumarate and α-ketoglutarate were significantly reduced after transfection of OE-*PER2*-SCC25 cells with sh-*PER2*#3 compared with OE-*PER2*-SCC25 cells. **I.** CCK-8 assay showed, cell proliferation levels were significantly increased in OE-PER2-SCC25 cells transfected with sh-*PER2*#3 compared with OE-PER2-SCC25 cells. **J.** MTT assay showed, cell proliferation levels were significantly increased in OE-PER2-SCC25 cells transfected with sh-*PER2*#3 compared with OE-PER2-SCC25 cells. All data represent three independent experiments. Data are expressed as means ± SD ($n \geq 3$). *$P < 0.05$; **$P < 0.01$; ***$P < 0.001$; ****$P < 0.0001$.

Figure S3

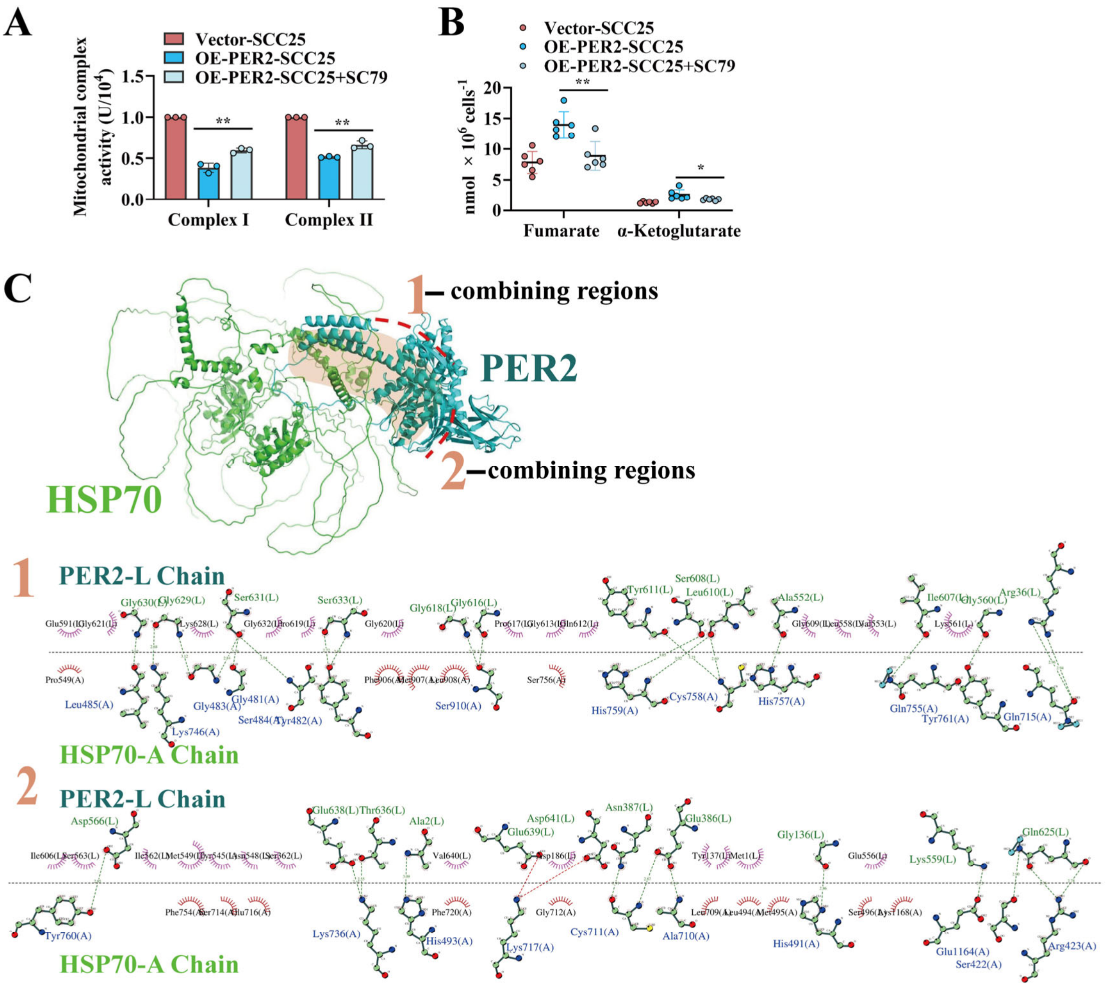

Figure S3. Protein–protein docking prediction

A. Micro-mitochondrial Complex I and II Activity Assay Kit showed a significant increase in electron transport chain complex I and II activity after addition of SC79 to OE-PER2-SCC25 cells. **B.** Fumarate Assay Kit and α-KG Assay Kit detected the concentration of fumarate and α-ketoglutarate was significantly reduced by the addition of SC79 to OE-PER2-SCC25 cells. **C.** Protein–protein docking predicted two binding sites for PER2 and HSP70 proteins, both of which are located in C-terminal structural domains of PER2 (L-chain for PER2, A-chain for HSP70, numbers denote peptide positions, letters denote amino acid abbreviations). All data represent three replicate independent experiments. Data are presented as mean ± SD. *$P < 0.05$; **$P < 0.01$; ***$P < 0.001$; ****$P < 0.0001$.

Figure S4

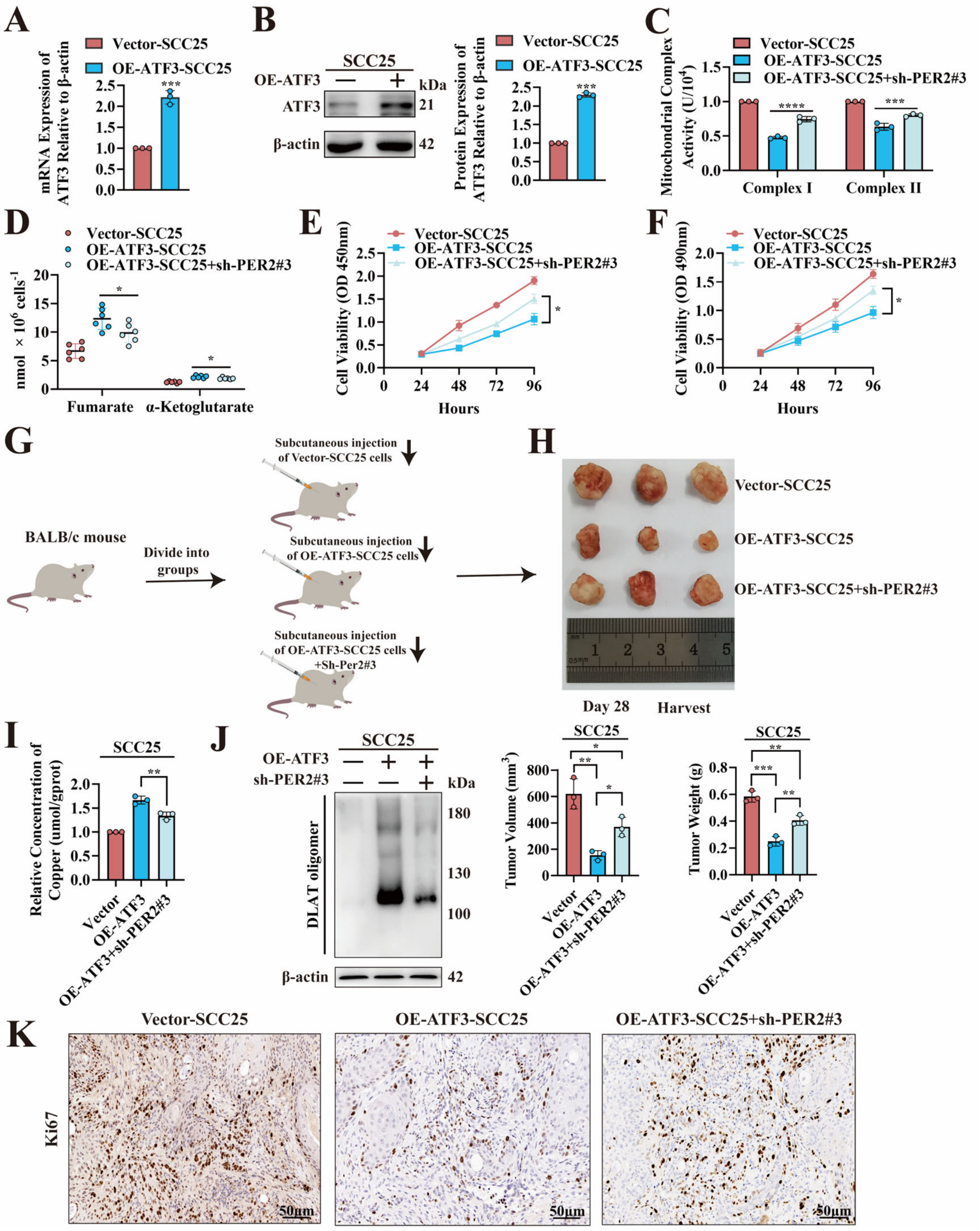

Figure S4. Construction of overexpressing *ATF3*-stable strains and ATF3 promotion of OSCC cuproptosis-dependent PER2

OE-ATF3-SCC25 cells with stable overexpression of *ATF3* was constructed in SCC25 cells with an *ATF3* overexpression efficiency of 2.29±0.07. Vector-SCC25 cells were used as a control. **A.** RT-qPCR showed, *ATF3* mRNA expression was significantly increased in OE-ATF3-SCC25 cells

compared with Vector-SCC25 cells. **B.** Western blotting showed, ATF3 protein expression was significantly increased in OE-ATF3-SCC25 cells compared with Vector-SCC25 cells. **C.** Micro-mitochondrial Complex I and II Activity Assay Kit detected mitochondrial electron transport chain complex I and II activity was significantly enhanced in OE-ATF3-SCC25+sh-*PER2*#3 cells compared with OE-ATF3-SCC25 cells. **D.** Fumarate Assay Kit and α-KG Assay Kit detected concentrations of fumarate and α-ketoglutarate were significantly lower in OE-ATF3-SCC25+sh-*PER2*#3 cells compared with OE-ATF3-SCC25 cells. **E.** CCK-8 assay showed, the proliferation level of OE-ATF3-SCC25+sh-*PER2*#3 cells was significantly enhanced compared with OE-ATF3-SCC25 cells. **F.** MTT assay showed, the proliferation level of OE-ATF3-SCC25+sh-*PER2*#3 cells was significantly enhanced compared with OE-ATF3-SCC25 cells. **G.** Schematic diagram of subcutaneous OSCC models established in BALB/c nude mice by subcutaneous inoculation of Vector-SCC25, OE-ATF3-SCC25 and OE-ATF3-SCC25+sh-*PER2*#3 cells. **H.** Subcutaneous tumor formation assay of nude mice: nude mice were injected subcutaneously with Vector-SCC25, OE-ATF3-SCC25, and OE-ATF3-SCC25+sh-*PER2*#3 cells, and the tumor weight and volume were measured after harvesting tumors on day 28. **I.** Copper Colorimetric Assay Kit for the detection of copper in tumors of Vector-SCC25, OE-ATF3-SCC25 and OE-ATF3-SCC25+ sh-*PER2*#3 groups. **J.** Non-denaturing gel electrophoresis assay for detecting DLAT oligomers in tumors of Vector-SCC25, OE-ATF3-SCC25 and OE-ATF3-SCC25+sh-*PER2*#3 groups. **K.** IHC assay for Ki67 expression in tumors of Vector-SCC25, OE-ATF3-SCC25 and OE-ATF3-SCC25+sh-*PER2*#3 groups (n = 3, scale bars = 50μm). All data represent three replicate independent experiments. Data are presented as mean ± SD. *$P < 0.05$; **$P < 0.01$; ***$P < 0.001$; ****$P < 0.0001$.

Figure S5

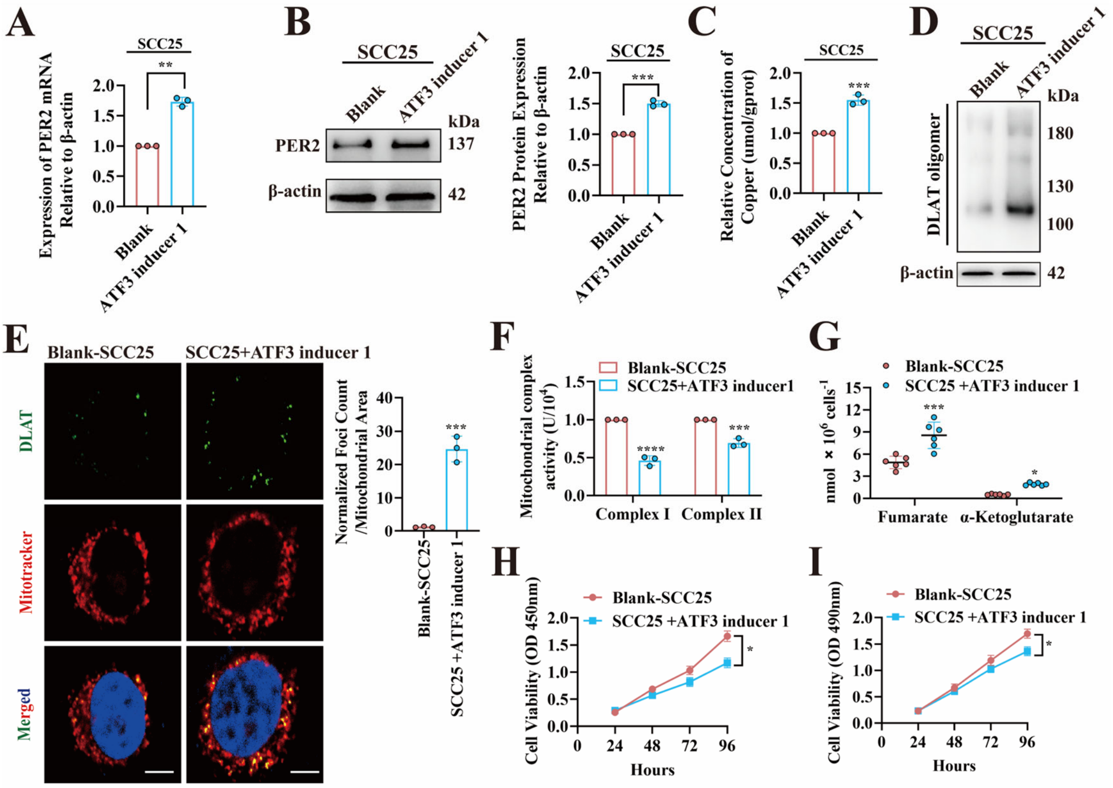

Figure S5. ATF3 inducer 1 targets upregulation of *PER2* to promote OSCC cuproptosis

The following assays were performed in SCC25 cells after the addition of ATF3 inducer 1. **A.** RT-qPCR for *PER2* mRNA expression. **B.** Western blotting for PER2 protein expression. **C.** Copper Colorimetric Assay Kit for copper in cells. **D.** Non-denaturing gel electrophoresis assay for DLAT oligomers in cells. **E.** Immunofluorescence assay for DLAT oligomers in cells (yellow, DLAT oligomer; green, DLAT; red, Mitotracker; blue, DAPI; scale bars = 50 μm; three independent experiments). **F.** Micro-mitochondrial Complex I and II Activity Assay Kit for determining mitochondrial electron transport complex I and II activity in cells. **G.** Fumarate Assay Kit and α-KG Assay Kit for measuring levels of fumarate and α-Ketoglutaric in cells. **H.** CCK-8 assay for determining levels of cell proliferation. **I.** MTT assay for detecting levels of cell proliferation. All data represent three replicate independent experiments. Data are presented as mean ± SD. **P* < 0.05; ***P* < 0.01; ****P* < 0.001; *****P* < 0.0001.
