# Supplementary material for: PER2 interaction with HSP70 promotes cuproptosis in oral squamous carcinoma cells by decreasing AKT stability
Source: Cell Death Dis. 2025 Mar 20;16(1):192. doi: 10.1038/s41419-025-07523-1 (PMC11926231; doi:10.1038/s41419-025-07523-1)
Supplement: Supplementary file 5 — Supplementary Tables S4-9 [file 41419_2025_7523_MOESM5_ESM.pdf]

# Supplementary Tables S4-9 for

**PER2 interaction with HSP70 promotes cuproptosis in oral squamous carcinoma cells by decreasing AKT stability**

Wenguang Yu[1], Shilin Yin[1], Hong Tang[1], Hengyan Li[1], Zhiwei Zhang[1], and Kai Yang[1]

**Table S4. sh-*PER2* sequences**

| ID | Accession | Target Seq |
|---|---|---|
| sh-PER2 #1 | NM_022817 | gcCAGAGTCCAGATACCTTTA |
| sh-PER2 #2 | NM_022817 | ccACGAGAATGAAATCCGCTA |
| sh-PER2 #3 | NM_022817 | ccAAATTGTTTGTTCCAGGAT |

**Table S5. Sequences of primers corresponding to each structural domain of overexpressed *PER2* and deletion mutant *PER2* containing Flag tags**

| ID | Forward sequence(5'to 3') | Reverse sequence(5' to 3') |
|---|---|---|
| Flag-PER2 | AGGTCGACTCTAGAGGATCCCGCCACC ATGAATGGATACGCGGAATTTCCGCCC | ACCGTAAGTTATGTGCTAGCTTACGT CTGCTCTTCGATCCTGTGATTC |
| Flag-Mut-PER2$^{\Delta PAS1}$ | GTTAAGCTTGGTACCGAGCTCGGA TCCgccaccATGAATGGATACGCG | CGCCACTGTGCTGGATATCTGCAGAA TTCCGTCTGCTCTTCGATCCTGT |
| Flag-Mut-PER2$^{\Delta PAS2}$ | GTTAAGCTTGGTACCGAGCTCGGA TCCgccaccATGAATGGATACGCG | CGCCACTGTGCTGGATATCTGCAGAA TTCCGTCTGCTCTTCGATCCTGT |
| Flag-Mut-PER2$^{\Delta CT}$ | GTTAAGCTTGGTACCGAGCTCGGATCC gccaccATGAATGGATACGCG | GCCACTGTGCTGGATATCTGCAGAAT TCGGAGGTCTGGCTCATAAGGT |

**Table S6. primer sequences for *PER2* promoter reporter gene plasmid, ATF3 plasmid, and *PER2* promoter truncation**

| ID | Forward sequence(5'to 3') | Reverse sequence(5' to 3') |
|---|---|---|
| pGL3-PER2 | GAGATTTGGTAAGAACAGCCCC | TGGAGCTCCCAAAGTTCGAC |
| OE-ATF3 | GGCTCAGAATGGGAGGA | GCTTCAGGGTTTTGGGTAT |
| Domain#1 | AGATGTCGAACTTTGGGAGC | GGCCAACCTAAGCGATTCCT |
| Domain#2 | AACACAGAAAGGCCAGTCCC | GGAAGCCAGCCGCAAAAATG |
| Domain#3 | CCAGGCCCTCACTCAGCA | TGGCACAGGGGCAGTCAT |

**Table S7. Primer sequences for *PER2* promoter point mutants**

| ID | Forward sequence(5'to 3') | Reverse sequence(5' to 3') |
|---|---|---|
| Mut | AACACAGAAAGGCCAGTCCC | GGAAGCCAGCCGCAAAAATG |

**Table S8. Primer sequences for RT-qPCR**

| Gene | Forward primer sequence (5’to 3’) | Reverse primer sequence (5’ to 3’) |
|---|---|---|
| PER2 | TATGGTGACTTCCCTTTGC | ATGTGATGTGGGCTTGG |
| FDX1 | TCATTACATGGTGGAAGGG | AATGCCGTTATCTCAGGTG |
| LIAS | TGGGTTTAGGCGAGAATG | GCCTTGTTGGCTGCATA |
| LIPT1 | CGGAGAAGAAGTGGAGGAG | GGGTTGGACAGCATTCAG |
| DLAT | ACCACTTTCCACCCCTTT | CACGCACGCTCTTTCTC |
| DLD | TGCCAACTTCCCTCTACTTT | ATATCAGACCAACCCTCCTG |
| PDHA1 | TCAACCCCACAGACCATC | CCCTTTAGCACAACCTCCT |
| PDHB | GCACCTGGATTTGTACTGC | GGGGAGGAGAGTGGAGAG |
| MTF1 | CTTTCTCTTGCCCTTTTCC | CCACCCTCCCCAAATAG |
| GLS | CTGTCCAGCTCTCCTTCG | CTCTTTGCCCTCGCTGT |
| CDKN2A | GACACGCTGGTGGTGCT | GCATGGTTACTGCCTCTGG |
| SLC31A1 | TGAGCCTACTGGATTGAGG | CCTTTGTGTTGGACTTCGT |
| ATP7A | GATCCAAGTATGGGTGTGAAT | AATCTGCTGCTCAATGGTC |
| ATP7B | TATGTCAGCCAGGTGTCG | GCCATTCAGGAGCAGAGA |
| β-actin | TCTCCCAAGTCCACACAGG | GGCACGAAGGCTCATCA |

**Table S9. Antibodies for Western Blotting**

| Antibody | Catalog NO. | Brand | Country | Dilution ratio | | | |
|---|---|---|---|---|---|---|---|
| | | | | IHC | WB | FC | Co-IP |
| PER2 | NB100-125SS | Novus | America | 1:200 | 1:1000 | | 1:100 |
| DLAT | T58125 | Abmart | China | 1:200 | 1:1000 | 1:50 | |
| PDHB | 14744-1-AP | Proteintech | America | 1:500 | 1:3000 | | |
| SLC31A1 | T510261 | Abmart | China | 1:500 | 1:1000 | | |
| HSP70 | M20033 | Abmart | America | | 1:3000 | | 1:100 |
| SDHB | 10620-1-AP | Proteintech | America | | 1:10000 | | |
| DPYD | 27662-1-AP | Proteintech | America | | 1:5000 | | |
| AKT | 4691 | CST | America | | 1:2000 | | 1:100 |
| p-AKT (473) | 4060 | CST | America | | 1:2000 | | 1:100 |
| Flag | 20543-1-AP | Proteintech | America | | 1:50000 | | 1:100 |
| Ubiquitin (P4D1) | 3936 | CST | America | | 1:1000 | | |
| ATF3 | ab207434 | Abcam | America | 1:100 | 1:1000 | | 1:50 |
| β-actin | 66009-1-Ig | Proteintech | America | | 1:30000 | | |
| Ki67 | 27309-1-AP | Proteintech | America | 1:5000 | | | |
| IgG | 30000-0-AP | Proteintech | America | | | | 1:100 |
| Anti-Rabbit IgG Alexa Fluor 488 | 4412S | CST | America | | | 1:1000 | |
