# Supplementary material for: PER2 interaction with HSP70 promotes cuproptosis in oral squamous carcinoma cells by decreasing AKT stability
Source: Cell Death Dis. 2025 Mar 20;16(1):192. doi: 10.1038/s41419-025-07523-1 (PMC11926231; doi:10.1038/s41419-025-07523-1)

Fig. 1J

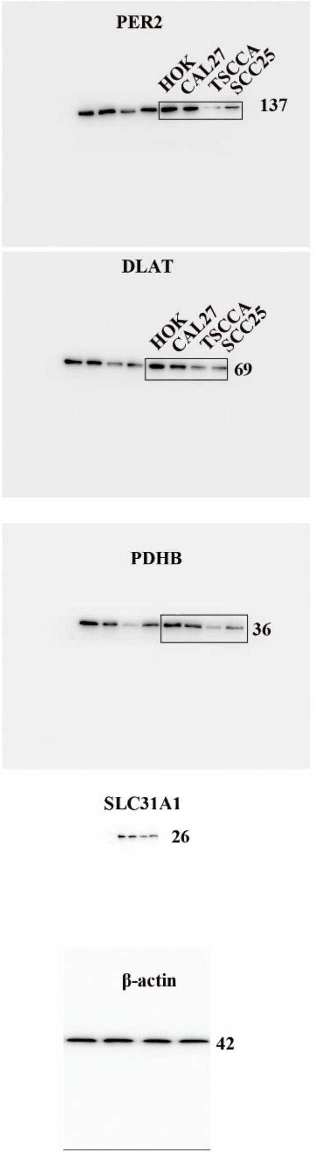
PER2
HOK
CAL27
TSCCA
SCC25
137
DLAT
HOK
CAL27
TSCCA
SCC25
69
PDHB
36
SLC31A1
26
β-actin
42

Fig. 2B

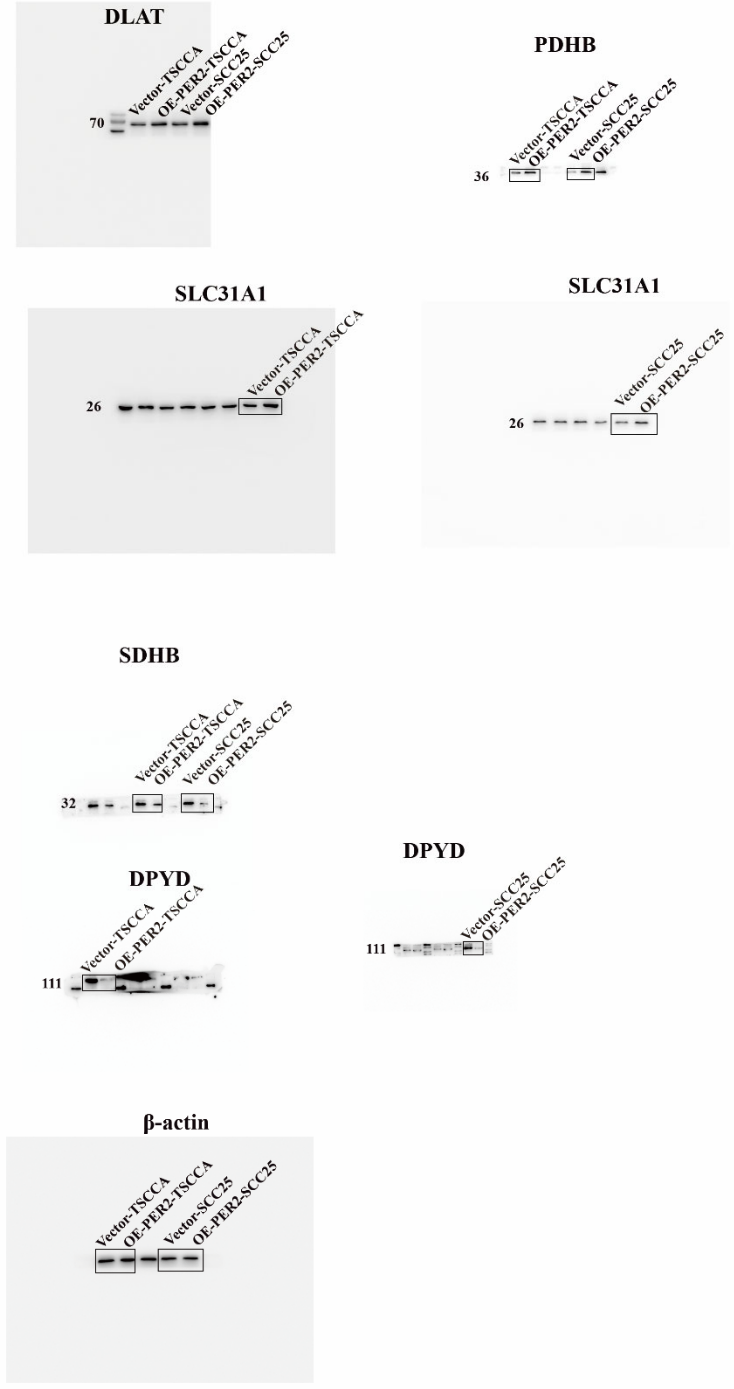
DLAT
Vector-TSCCA
OE-PER2-TSCCA
Vector-SCC25
OE-PER2-SCC25
70
PDHB
Vector-TSCCA
OE-PER2-TSCCA
Vector-SCC25
OE-PER2-SCC25
36
SLC31A1
Vector-TSCCA
OE-PER2-TSCCA
26
SLC31A1
Vector-SCC25
OE-PER2-SCC25
26
SDHB
Vector-TSCCA
OE-PER2-TSCCA
Vector-SCC25
OE-PER2-SCC25
32
DPYD
Vector-TSCCA
OE-PER2-TSCCA
111
DPYD
Vector-SCC25
OE-PER2-SCC25
111
β-actin
Vector-TSCCA
OE-PER2-TSCCA
Vector-SCC25
OE-PER2-SCC25

Fig. 2D

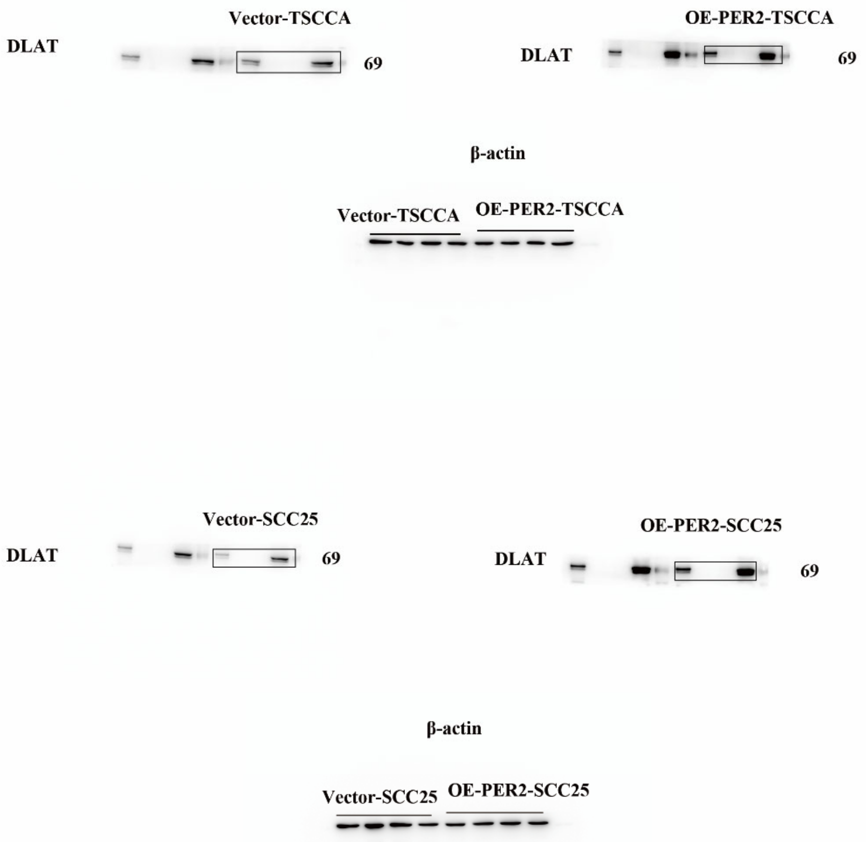
Vector-TSCCA
OE-PER2-TSCCA
DLAT
69
DLAT
69
β-actin
Vector-TSCCA
OE-PER2-TSCCA
Vector-SCC25
OE-PER2-SCC25
DLAT
69
DLAT
69
β-actin
Vector-SCC25
OE-PER2-SCC25

Fig. 2E

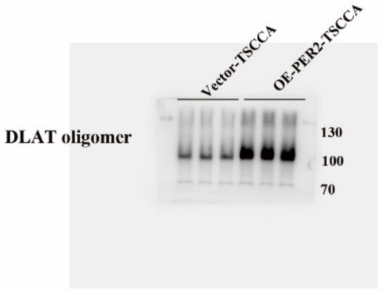
Vector-TSCCA
OE-PER2-TSCCA
DLAT oligomer
130
100
70

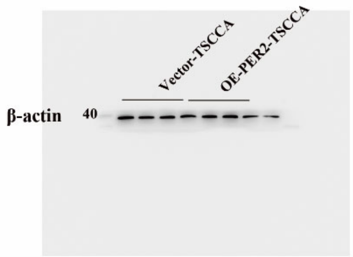
Vector-TSCCA
OE-PER2-TSCCA
β-actin
40

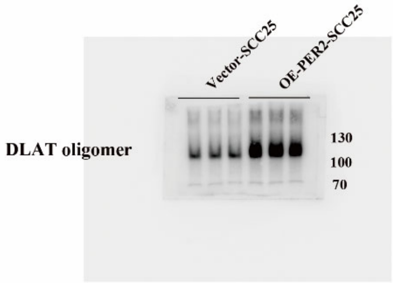
Vector-SCC25
OE-PER2-SCC25
DLAT oligomer
130
100
70

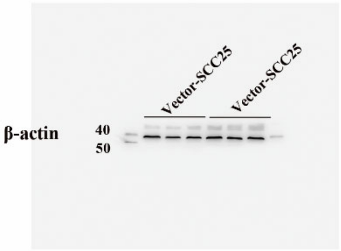
Vector-SCC25
Vector-SCC25
β-actin
40
50

Fig. 3C

PER2

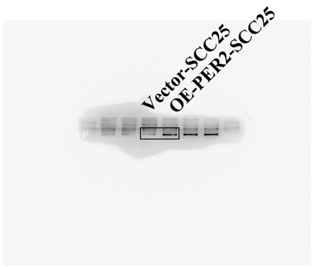

DLAT

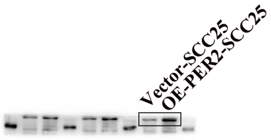

PDHB

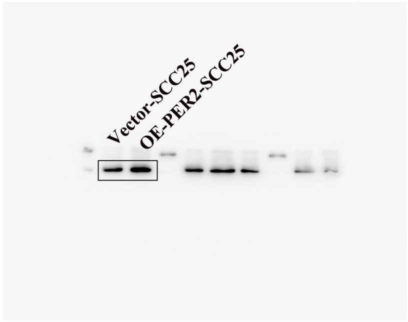

SLC31A1

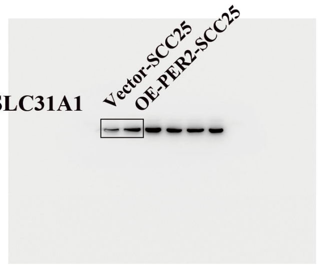

β-actin

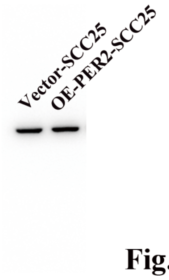

Fig. 3E

DLAT oligomer

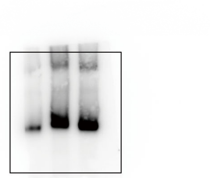

β-actin

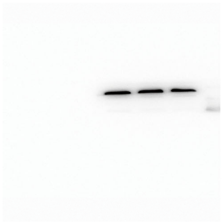

Fig. 4A

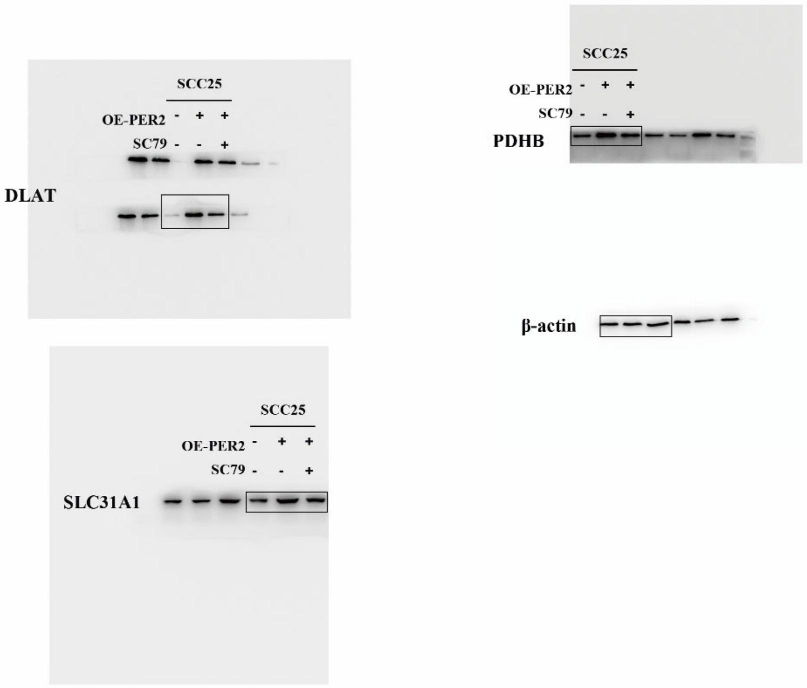
SCC25
OE-PER2 - + +
SC79 - - +
DLAT
SCC25
OE-PER2 - + +
SC79 - - +
PDHB
β-actin
SCC25
OE-PER2 - + +
SC79 - - +
SLC31A1

Fig. 4C

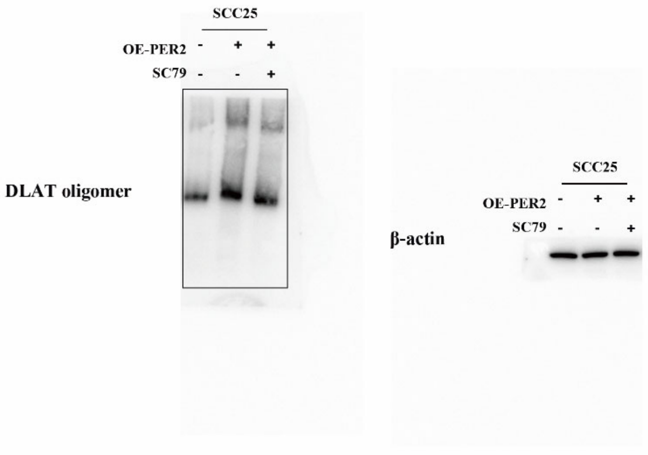
SCC25
OE-PER2 - + +
SC79 - - +
DLAT oligomer
SCC25
OE-PER2 - + +
SC79 - - +
β-actin

Fig. 4H

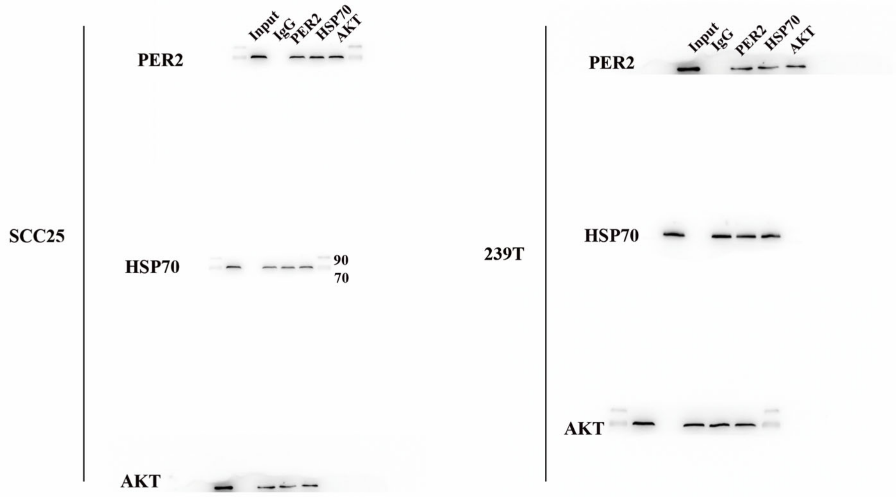
SCC25
Input
IgG
PER2
HSP70
AKT
PER2
HSP70
90
70
AKT
239T
Input
IgG
PER2
HSP70
AKT
PER2
HSP70
AKT

Fig. 4I

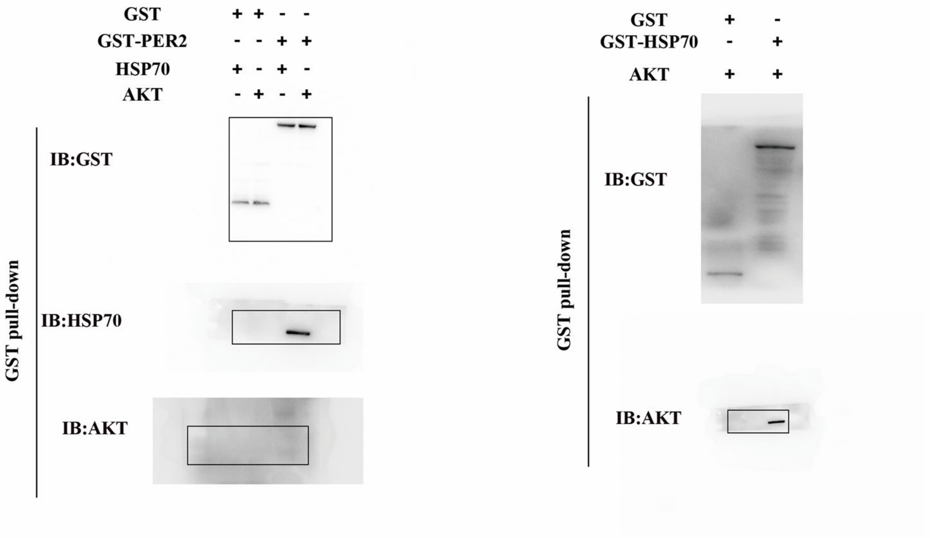
GST + + - -
GST-PER2 - - + +
HSP70 + - + -
AKT - + - +
GST pull-down
IB:GST
IB:HSP70
IB:AKT
GST + -
GST-HSP70 - +
AKT + +
GST pull-down
IB:GST
IB:AKT

Fig. 4L

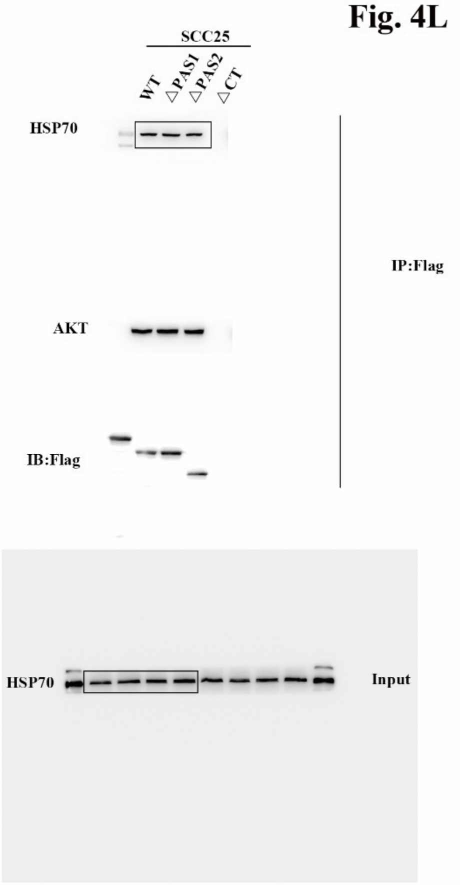

Fig. 5A

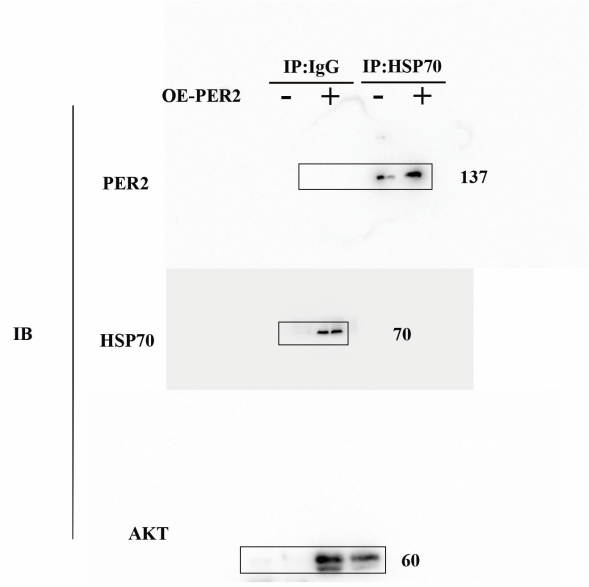
IP:IgG
IP:HSP70
OE-PER2
-
+
-
+
PER2
137
IB
HSP70
70
AKT
60

Fig. 5B

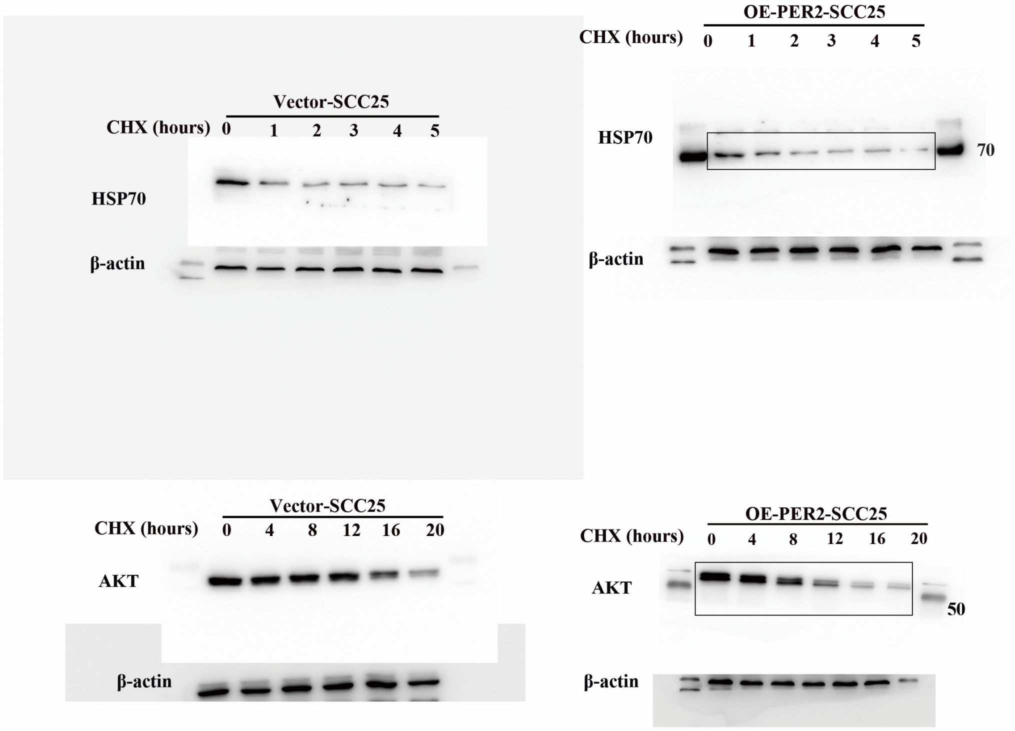
Vector-SCC25
CHX (hours) 0 1 2 3 4 5
HSP70
β-actin
OE-PER2-SCC25
CHX (hours) 0 1 2 3 4 5
HSP70
70
β-actin
Vector-SCC25
CHX (hours) 0 4 8 12 16 20
AKT
β-actin
OE-PER2-SCC25
CHX (hours) 0 4 8 12 16 20
AKT
50
β-actin

Fig. 5C

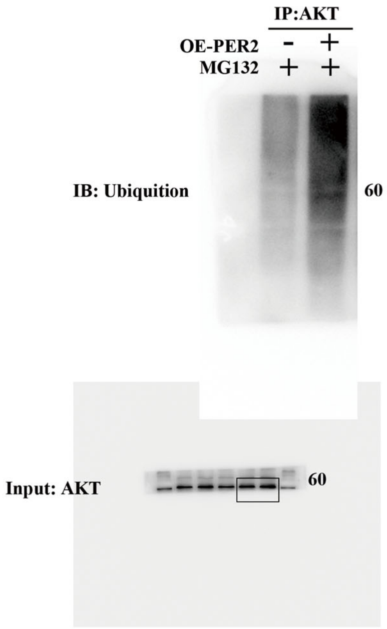

Fig. 5D

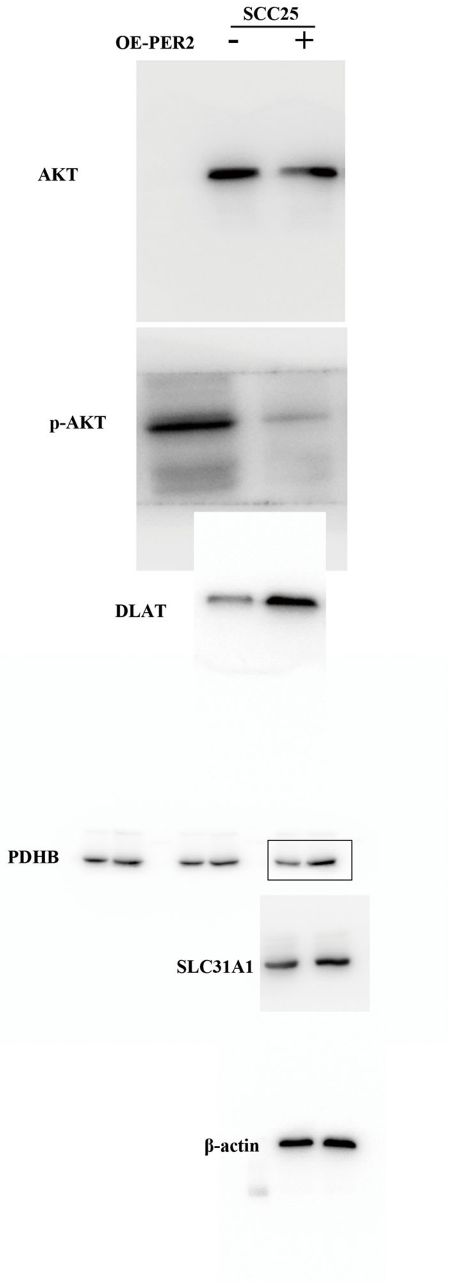

Fig. 5F

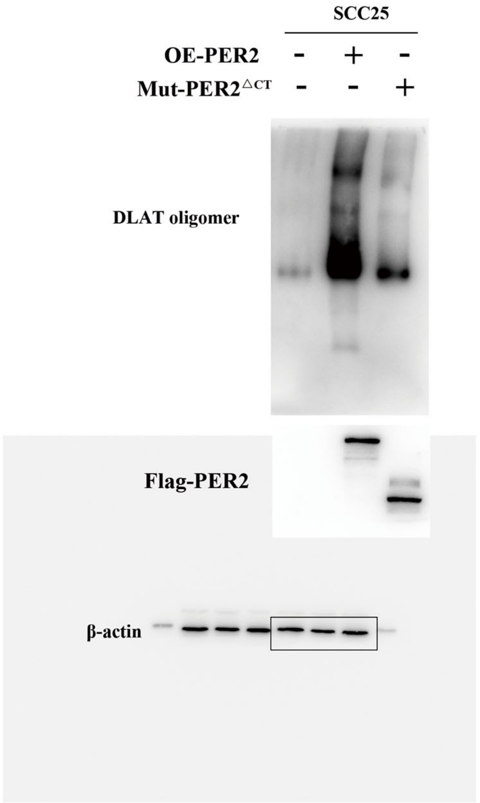

Fig. 5L

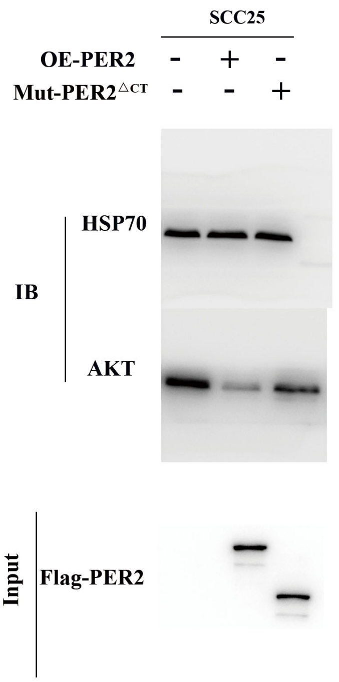
SCC25
OE-PER2 - + -
Mut-PER2△CT - - +
IB
HSP70
AKT
Input
Flag-PER2

Fig. 5M

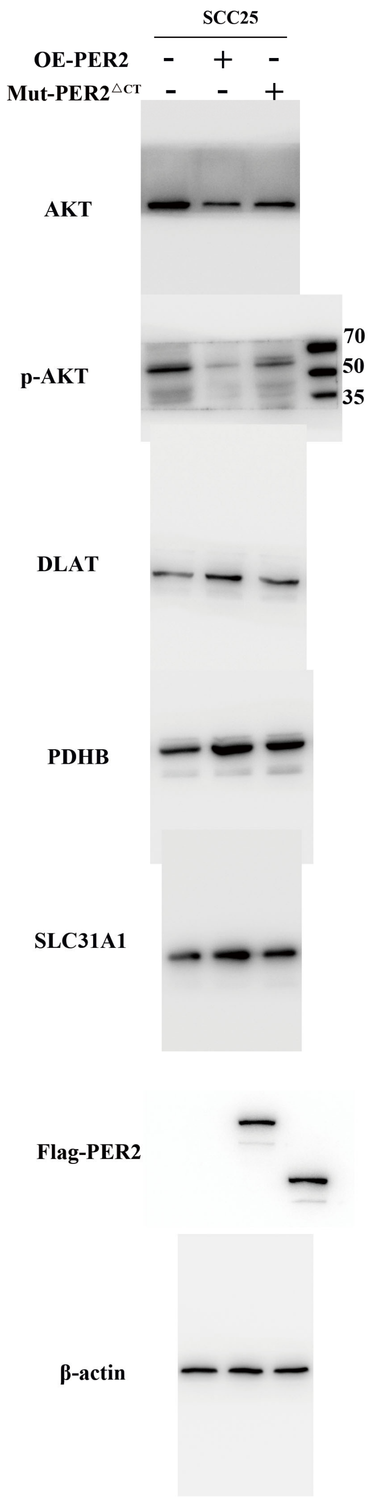
SCC25
OE-PER2 - + -
Mut-PER2△CT - - +
AKT
p-AKT
70
50
35
DLAT
PDHB
SLC31A1
Flag-PER2
β-actin

Fig. 6C

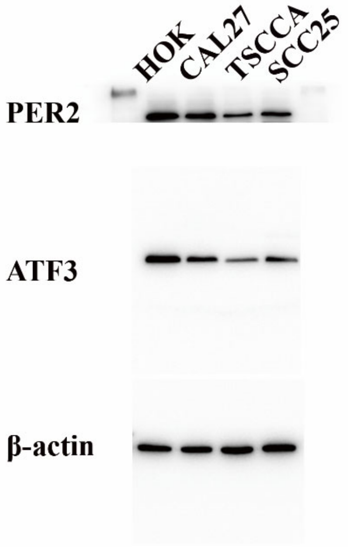

Fig. 7B

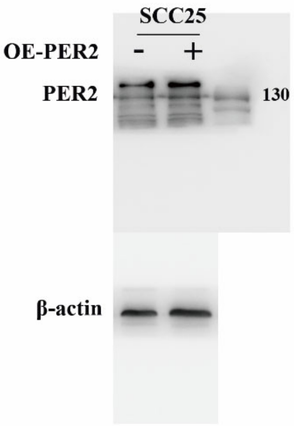

Fig. 7D

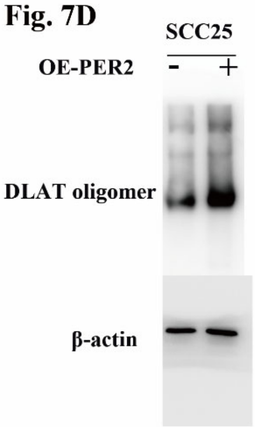

Fig. 7L

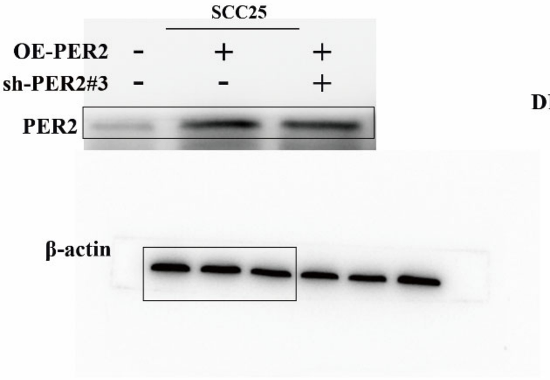

Fig. 7M

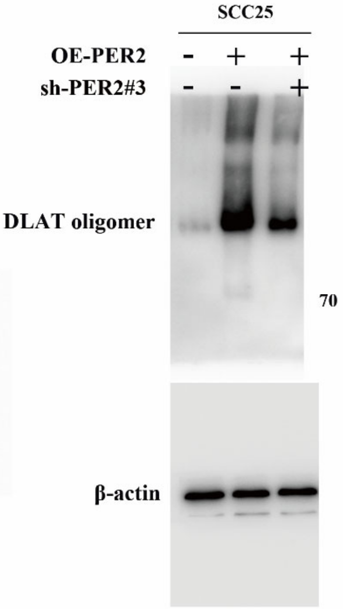

Fig. 8C

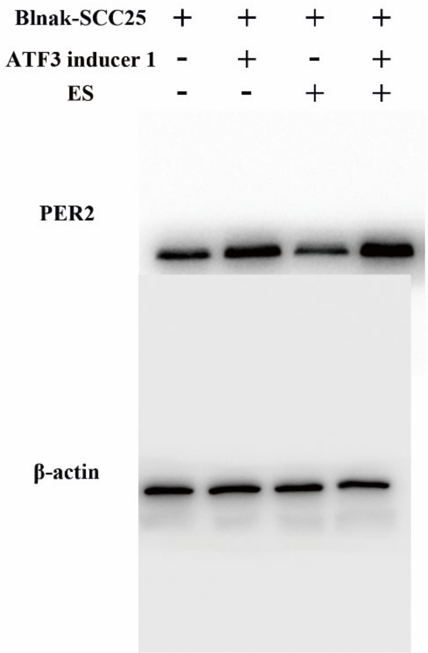

Blnak-SCC25 + + + +
ATF3 inducer 1 - + - +
ES - - + +
PER2
β-actin

Fig. 8E

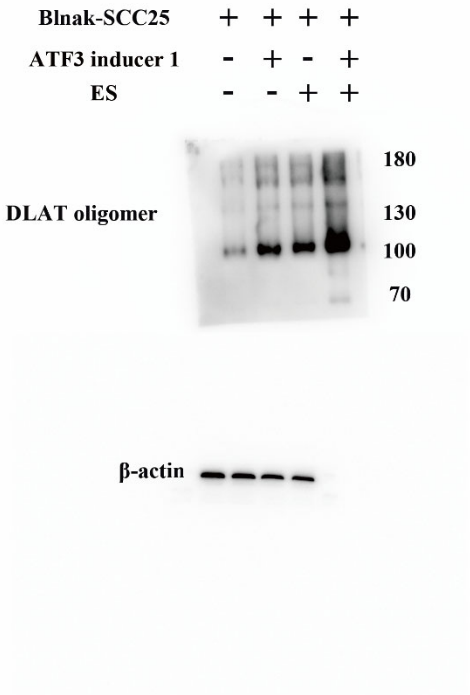

Blnak-SCC25 + + + +
ATF3 inducer 1 - + - +
ES - - + +
DLAT oligomer
180
130
100
70
β-actin

Fig. S1B

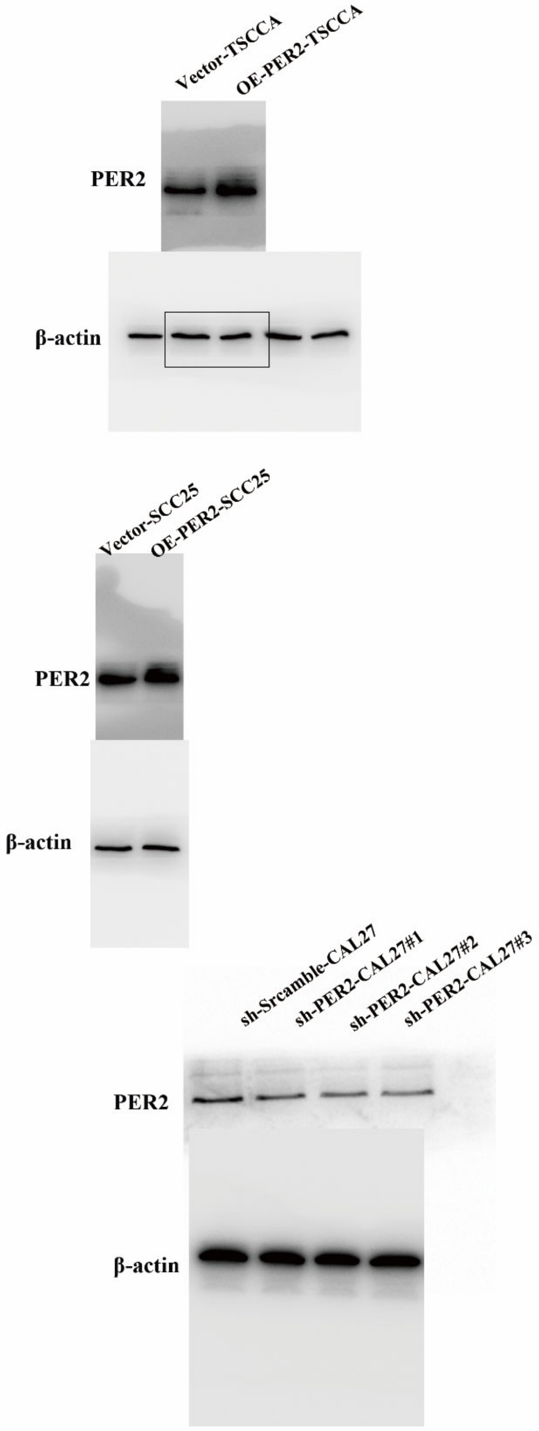

Fig. S1D

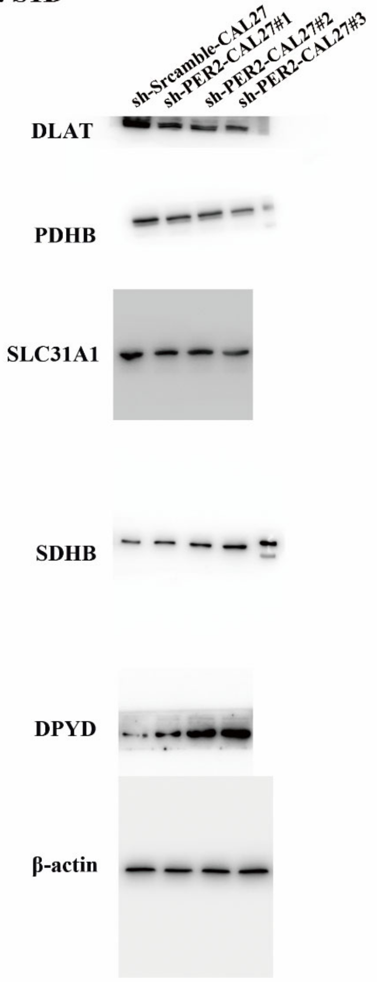

Fig. S2B

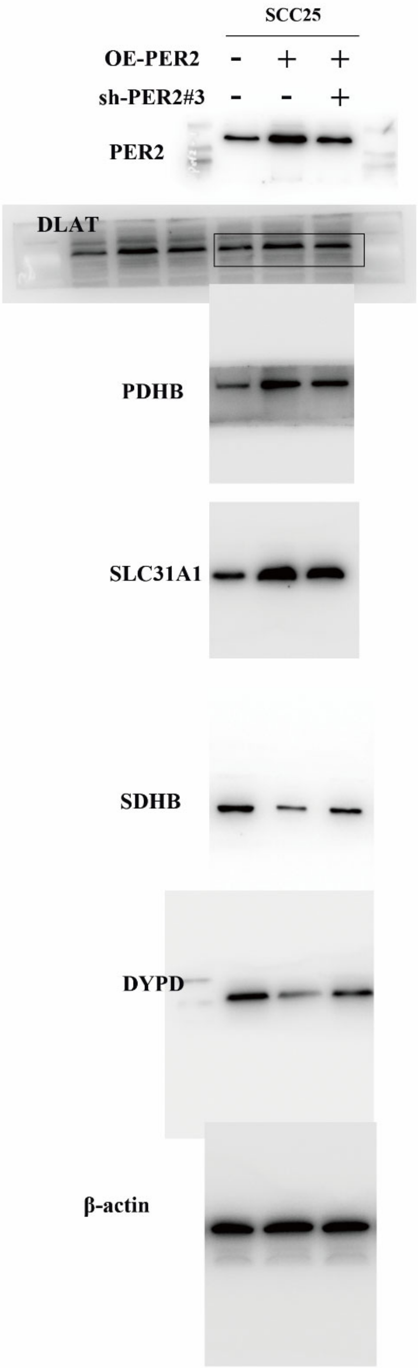

Fig. S2D

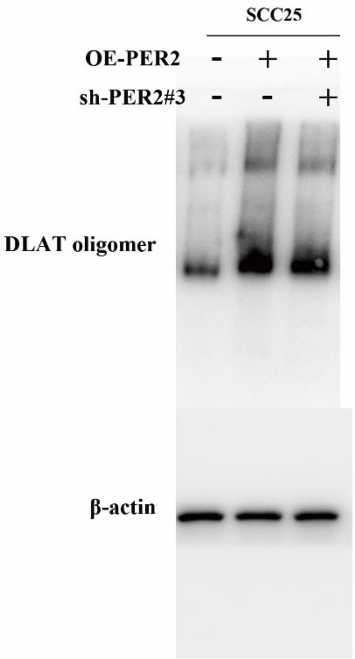

Fig. S4B

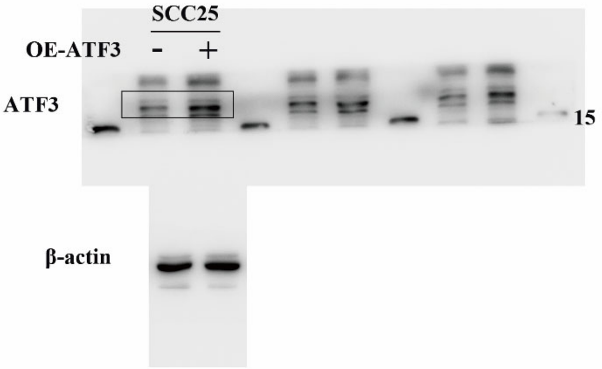

Fig. S4J

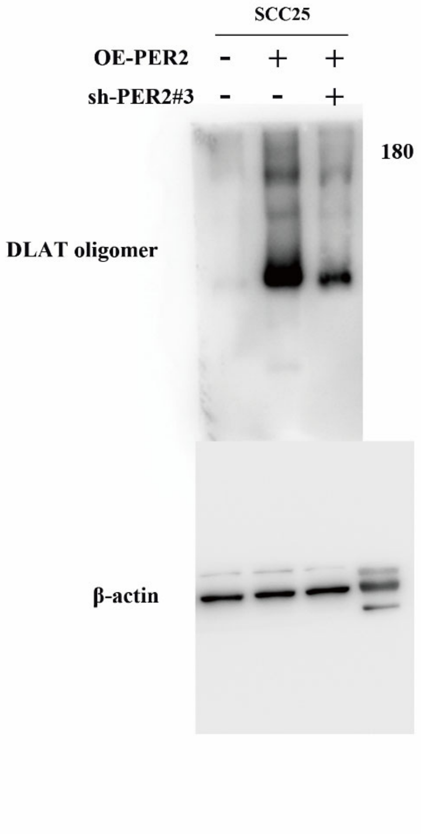

Fig. S5B

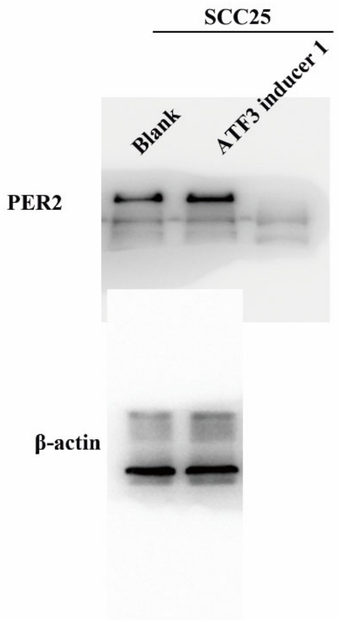

Fig. S5D

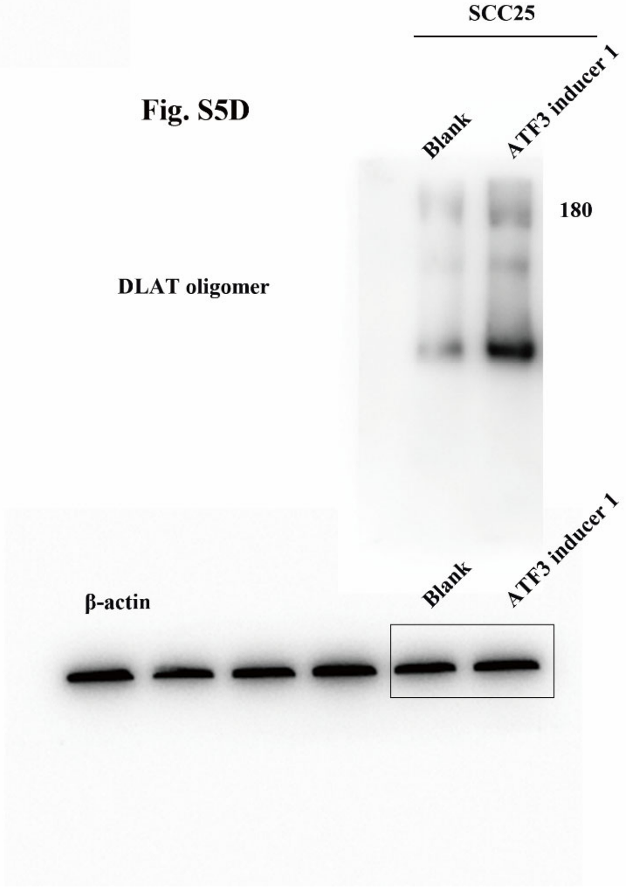

Supplement: Supplementary file 6 — Original western blot images [file 41419_2025_7523_MOESM6_ESM.pdf]
